# Supplementary material for: Evaluation of NEWS2 response thresholds in a retrospective observational study from a UK acute hospital
Source: BMJ Open. 2022 Feb 8;12(2):e054027. doi: 10.1136/bmjopen-2021-054027 (PMC8830252; doi:10.1136/bmjopen-2021-054027)
Supplement: Supplementary data [file bmjopen-2021-054027supp001.pdf]

An Evaluation of NEWS2 response thresholds in a retrospective observational study from a UK acute hospital

Online supplement.

Dr Tanya Pankhurst, Dr Elizabeth Sapey, Ms Helen Gyves, Ms Felicity Evison, Ms Suzy Gallier, Professor Georgios Gkoutos, Professor Simon Ball

Online Supplementary Figure 1 (Figure S1)

Post admission days with no Index NEWS2 recorded

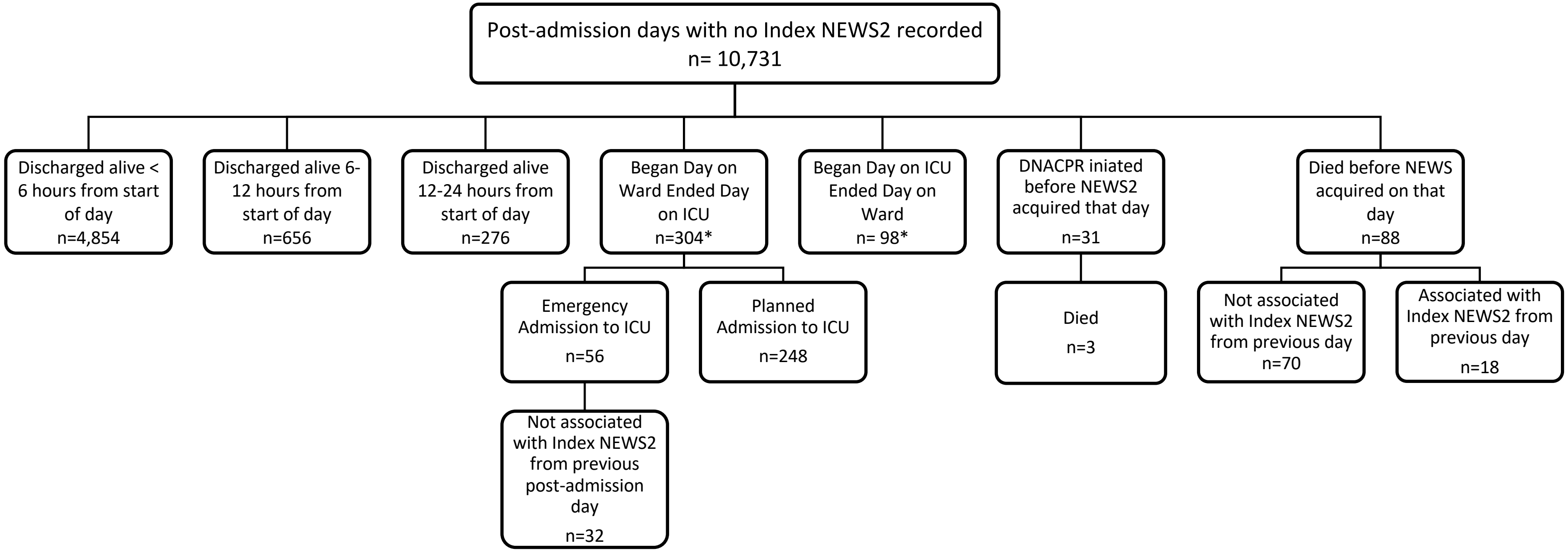

Online Supplementary Figure 1 (Fig S1)

Post admission days with no Index NEWS2 recorded

Of the 10,731 in which no Index-NEWS2 was recorded, 5,786 (53.9%) were discharged alive later that day. 433 (4.0%) were ineligible for part of the day because they were on ICU or had a DNACPR initiated. A further 88 (0.8%) died before a NEWS2 was recorded on that day. 4,424 post-admission days were therefore not associated with a NEWS2 record for unidentified reasons.

There were 144 events (88 deaths and 56 emergency admissions to ICU) associated with post-admission days in which no Index-NEWS2 was recorded and another 3 in which a DNACPR was initiated prior to NEWS2 acquisition and subsequent death. Of the 144 events, 102 were not associated with an Index NEWS2 from the previous day, 20 because they occurred on the first day. The other 82 were associated with NEWS2 acquired later in the previous day but occurred > 24 hours after that day's Index-NEWS2.

Online Supplementary Figure 2a (Fig S2a)

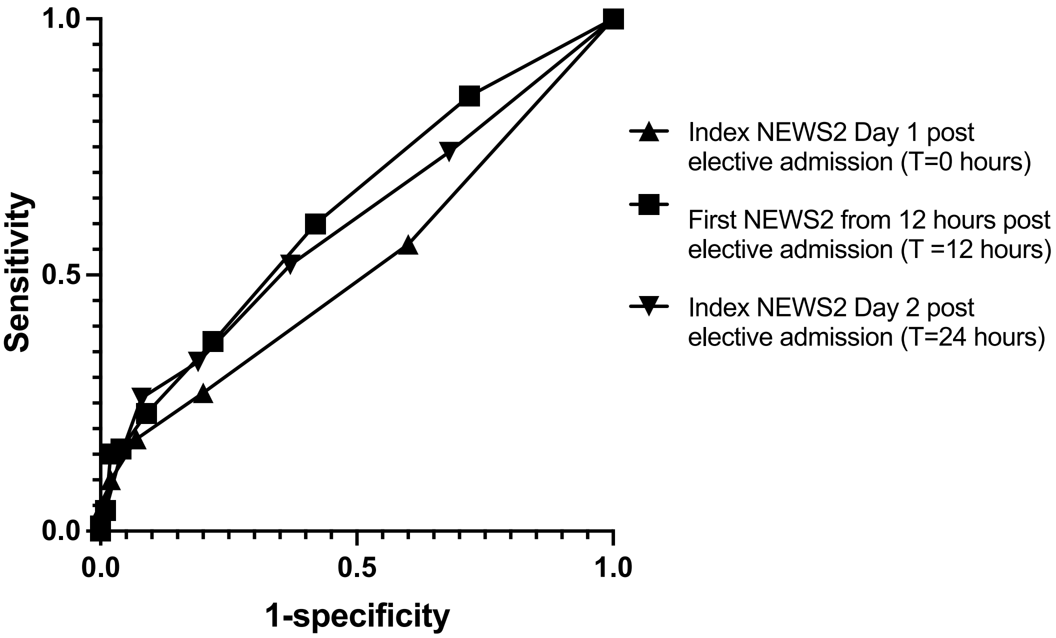

Legend Figure S2a

Receiver operating characteristic (ROC) for the first NEWS2 obtained from 0-, 12- and 24-hours post elective-admission. The respective c-statistics were 0.51, 0.63 and 0.60. The c-statistic = 0.74 for all later post elective admission patient-days combined. The outcome event was the first of unplanned admission to ICU (type 1 and 2 of the NHS critical care minimum dataset) or death of the patient within 24 hours of an evaluated NEWS2 score. As anticipated in the study design, Index NEWS2 on Day 1 of elective admission was not predictive of an adverse outcome. Its acquisition almost always preceded physiological insult, specifically a planned procedure occurring in 94.7% of patients in the subsequent 12 hours. Assessment of the discriminatory power of NEWS2 in the hours immediately following a procedure is beyond the scope of this analysis but appears low.

The NEWS2 associated event rate on Day 1 post elective-admission = 0.37/100 patient-days and Day 2 = 0.34/100 patient-days.

Online Supplementary Figure 2b (Fig S2b)

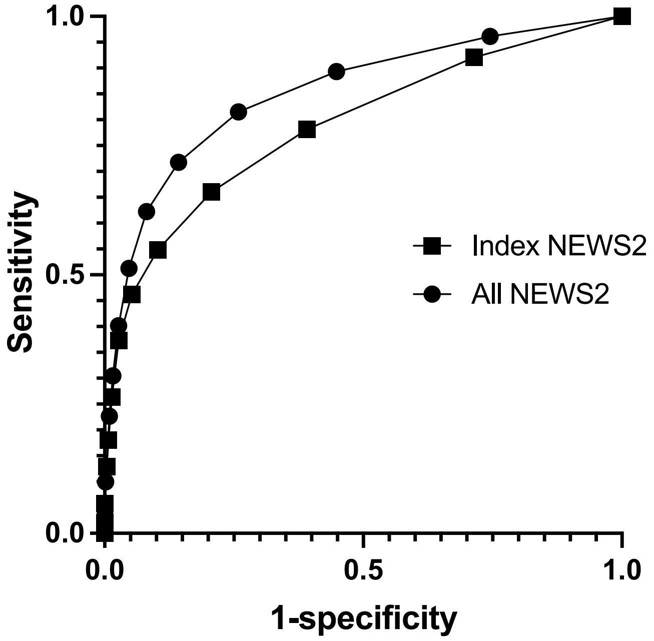

Legend Figure S2b

Receiver operating characteristic (ROC) for Index NEWS2 recorded in Day 1 to 56 excluding first post elective admission day (c statistic = 0.78, 95% CI:0.76-0.81) and All NEWS2 recorded in Days 1 to 56 excluding first post elective admission day (c statistic = 0.85, 95% CI: 0.85-0.86) p<0.001. A bootstrap analysis was performed with 10,000 repetitions of 10,000 patient-days, the median and 2.5th and 97.5th percentiles were then used to generate the ROC curve and corresponding confidence intervals. The outcome event was defined as the first of unplanned admission to ICU (type 1 and 2 of the NHS critical care minimum dataset) or death of the patient within 24 hours of a NEWS2 score.

Online Supplementary Figure 3a (Fig S3a)

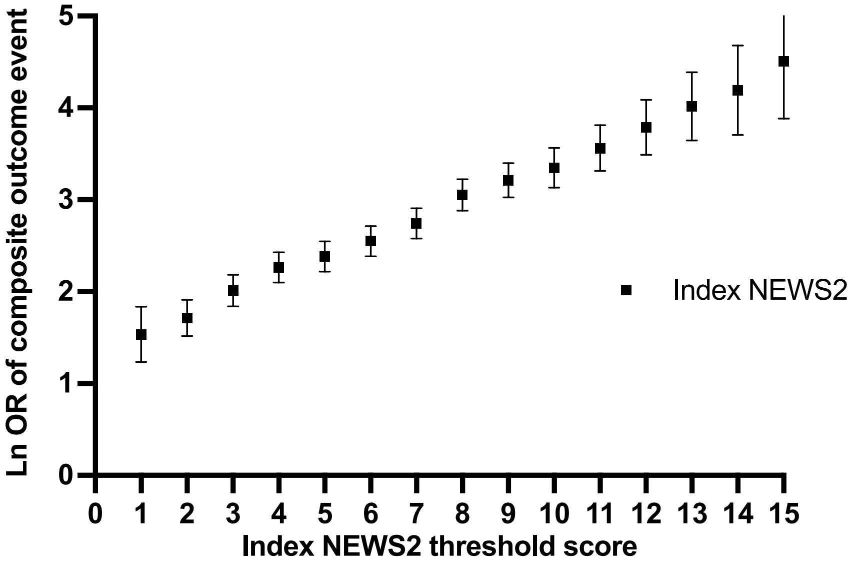

Legend Figure S3a

The Ln of the Odds Ratio (Ln OR) ± 95% confidence intervals for occurrence of the composite outcome event when the Index NEWS2 ≥ threshold score *vs.* < threshold score. The composite outcome event was the first of unplanned admission to ICU (type 1 and 2 of the NHS critical care minimum dataset) or death of the patient within 24 hours of a NEWS2 score. This analysis included NEWS2 scores from Day 1 to 56 post-admission other than for Day 1 of elective admission (Table S2a and S2b).

Although NEWS2 is not constituted to report absolute risk, it exhibits features useful in representing risk. Integer changes in threshold were associated with an approximately equal change in the odds ratio of an outcome event. Across the range of scores, the OR of an event ≥ threshold score *vs.* < threshold score, increased by a factor of 1.2. (Ln OR of composite outcome event = 1.35 + 0.205 (Index NEWS2 threshold score); R^2 = 0.99. For increase in NEWS2 =1, the increase in Ln OR of composite outcome event = 0.205, the OR of composite outcome event increases by a factor of $e^{0.205}$ = 1.2).

(These relationships resemble Fig 2b, because the relationship between sensitivity and Index NEWS2 is approximately negative linear and because at low event rates the odds of an event at threshold ≈ PPV = 1/NNE; the Ln (odds of an event) ≈ -Ln (NNE)).

Online Supplementary Figure 3b (Fig S3b)

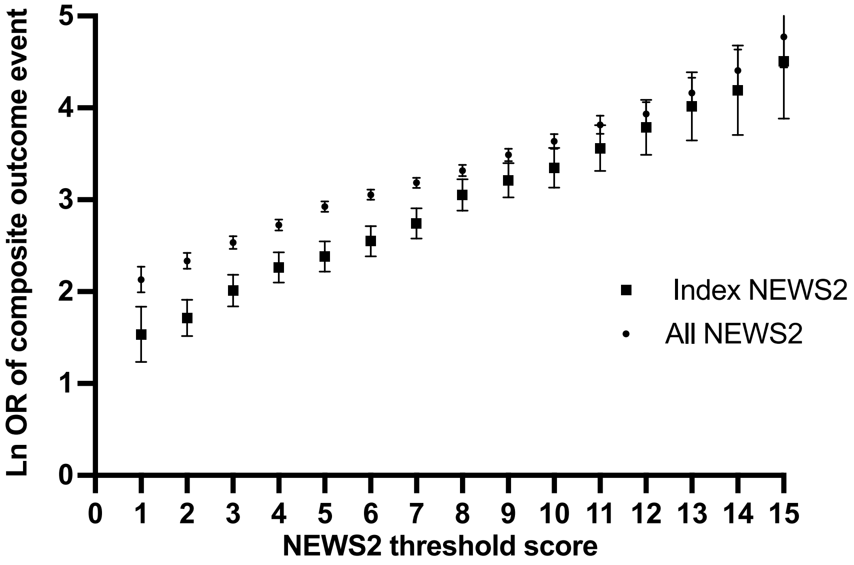

Legend Figure S3b

The Ln of the Odds Ratio (Ln OR) ± 95% confidence intervals for occurrence of the composite outcome event when the Index NEWS2 or All NEWS2 ≥ threshold score *vs.* < threshold score. This representation of the data reflects the differences in performance between Index-and All-NEWS2 shown in Fig S2b.

Supplementary Table S1 NEWS2 and SEWS scoring

National Early Warning Score (NEWS) 2 Standardising the assessment of acute-illness severity in the NHS– Royal College Physicians, London

Chart1: The NEWS2 scoring system

Physiological parameter				Score			
	3	2	1	0	1	2	3
Respiration rate (per minute)	≤8		9–11	12–20		21–24	≥25
SpO_2 Scale 1 (%)	≤91	92–93	94–95	≥96			
SpO_2 Scale 2 (%)	≤83	84–85	86–87	88–92 ≥93 on air	93–94 on oxygen	95–96 on oxygen	≥97 on oxygen
Air or oxygen?		Oxygen		Air			
Systolic blood pressure (mmHg)	≤90	91–100	101–110	111–219			≥220
Pulse (per minute)	≤40		41–50	51–90	91–110	111–130	≥131
Consciousness				Alert			CVPU
Temperature (°C)	≤35.0		35.1-36.0	36.1-38.0	38.1-39.0	≥39.1	

Chart 2: NEWS2 thresholds and triggers

NEW score Clinical risk Response		
Aggregate score 0–4	Low	Ward-based response
Red score<br>Score of 3 in any individual parameter	Low–medium	Urgent ward-based response*
Aggregate score 5–6	Medium	Key threshold for urgent response*
Aggregate score 7 or more	High	Urgent or emergency response**

* Response by a clinician or team with competence in the assessment and treatment of acutely ill patients and in recognising when the escalation of care to a critical care team is appropriate.
**The response team must also include staff with critical care skills, including airway management.
National Early Warning Score (NEWS) 2 Standardising the assessment of acute-illness severity in the NHS– Royal College Physicians, London,
© Royal College of Physicians 2017

Standardised early warning scoring system (SEWS) parameters and scoring system.

Physiological parameter				Score			
	3	2	1	0	1	2	3
Respiration rate (per minute)	≤8			9–20	21-30	31-35	≥36
SaO_2	<85	85-89	90-92	≥93			
Temperature (°C)	≤33.9	34.0-34.9	35.0-35.9	36.0-37.9	38.0-38.9	≥39.0	
Blood pressure (mmHg)	≤69	70-79	80-99	100-199		≥200	
Pulse (per minute)	≤29	30-39	40-49	50-99	100-109	110-129	≥130
AVPU				Alert	Verbal	Pain	None

Supplementary Table S2 – Index NEWS2 and SEWS (a) and linked outcomes events (b) from Day 1 to 56 post-admission excluding Day 1 of elective admission

Table S2a Index NEWS2 and SEWS

		NEWS2																				
		0	1	2	3	4	3 single	4 single	5	6	7	8	9	10	11	12	13	14	15	16	17	Total SEWS
SEWS	0	72852	54298	21005	6960	2310	123	110	891	232	40	5	1	1	0	0	0	0	0	0	0	158828
	1	674	28195	22333	10054	3685	2628	1565	2212	867	292	57	19	3	1	1	0	0	0	0	0	72586
	2	283	835	3995	4771	2162	1029	1512	1910	952	427	174	60	16	3	1	0	0	0	0	0	18130
	3	2	170	291	800	925	445	478	1068	793	481	246	102	35	17	4	0	0	0	0	0	5857
	4	0	5	27	43	156	9	123	394	431	301	211	143	68	15	7	2	2	0	0	0	1937
	5	0	0	0	4	10	3	14	62	129	143	157	118	85	39	16	4	6	0	1	0	791
	6	0	0	0	0	0	0	0	5	21	40	66	66	59	46	23	10	2	0	0	0	338
	7	0	0	0	0	0	0	0	0	1	5	15	27	24	26	12	8	2	2	1	0	123
	8	0	0	0	0	0	0	0	0	2	0	6	7	7	10	10	5	4	3	1	1	56
	9	0	0	0	0	0	0	0	0	0	0	0	2	2	2	4	4	2	2	0	0	18
	10	0	0	0	0	0	0	0	0	0	0	0	0	0	2	1	2	1	0	1	2	9
	11	0	0	0	0	0	0	0	0	0	0	0	0	0	1	0	1	0	0	1	0	3
	12	0	0	0	0	0	0	0	0	0	0	0	0	0	0	0	0	0	1	0	0	1
	13	0	0	0	0	0	0	0	0	0	0	0	0	0	0	0	0	0	0	0	1	1
	Total	73811	83503	47651	22632	9248	4237	3802	6542	3428	1729	937	545	300	162	79	36	19	8	5	4	
	Cum Total	73765	157187	204768	231572	244572			251062	254427	256108	257015	257536	257818	257966	258038	258068	258083	258090	258094	258098	

Of the 8039 that NEWS2 = 3 in any single parameter 149 (1.8%) triggered the lowest SEWS threshold ≥4
Of the 6542 that NEWS2 = 5, 461 triggered (7.0%) the lowest SEWS threshold ≥4
Of the 3428 that NEWS2 = 6, 584 triggered (16.0%) the lowest SEWS threshold ≥4
Cum Total Is the cumulative total at or below the NEWS2 score *without an event* used to calculate the odds ratio of the composite outcome event when the Index NEWS2 ≥ threshold score *vs.* < threshold score (Fig S3a).

Table S2b outcome events linked to Index NEWS2 and SEWS

		NEWS2																				
		0	1	2	3	4	3 single	4 single	5	6	7	8	9	10	11	12	13	14	15	16	17	Total SEWS
SEWS	0	45	52	28	21	9	0	0	3	2	1	0	0	0	0	0	0	0	0	0	0	161
	1	0	25	30	19	19	5	3	12	18	5	0	1	0	0	0	0	0	0	0	0	137
	2	1	3	11	11	7	2	5	20	16	6	5	3	1	0	1	0	0	0	0	0	92
	3	0	1	1	4	5	3	1	14	16	20	8	7	0	0	1	0	0	0	0	0	81
	4	0	0	0	0	0	0	1	3	8	7	5	4	1	1	0	0	0	0	0	0	30
	5	0	0	0	0	0	0	0	0	2	8	9	6	8	4	3	0	2	0	0	0	42
	6	0	0	0	0	0	0	0	0	1	1	3	1	6	4	2	1	0	0	0	0	19
	7	0	0	0	0	0	0	0	0	0	0	0	0	2	5	0	2	0	0	0	0	9
	8	0	0	0	0	0	0	0	0	0	0	0	2	0	0	0	1	2	1	1	0	7
	9	0	0	0	0	0	0	0	0	0	0	0	0	0	0	0	1	0	0	0	0	1
	10	0	0	0	0	0	0	0	0	0	0	0	0	0	0	0	1	0	0	0	0	1
	11	0	0	0	0	0	0	0	0	0	0	0	0	0	0	0	0	0	0	0	0	0
	12	0	0	0	0	0	0	0	0	0	0	0	0	0	0	0	0	0	0	0	0	0
	13	0	0	0	0	0	0	0	0	0	0	0	0	0	0	0	0	0	0	0	0	0
	Total	46	81	70	55	40	10	10	52	63	48	30	24	18	14	7	6	4	1	1	0	
	Cum Total	46	127	197	262	312			364	427	475	505	529	547	561	568	574	578	579	580	580	

Of the 20 that NEWS2 = 3 in any single parameter, 1 (5.0%) triggered the lowest SEWS threshold ≥4
Of the 52 that NEWS2 = 5, 3 (5.8%) the lowest SEWS threshold ≥4
Of the 63 that NEWS2 = 6, 11 triggered (17.5%) the lowest SEWS threshold ≥4
Cum Total Is the cumulative total at or below the NEWS2 score *with an event* used to calculate the odds ratio of the composite outcome event when the Index NEWS2 ≥ threshold score *vs.* < threshold score (Fig S3a).

Supplementary Table S3 – All NEWS2 and SEWS (a) and linked outcomes events (b) from Day 1 to 56 post-admission excluding Day 1 of elective admission

Table S3a All NEWS2 and SEWS

		NEWS2																				
		0	1	2	3	4	3 single	4 single	5	6	7	8	9	10	11	12	13	14	15	16	17	18	Total SEWS
	0	291135	225784	99131	37585	12886	631	500	4720	1444	269	46	11	1	0	0	0	0	0	0	0	0	674143
	1	2748	114393	99833	49029	20759	12435	7958	13629	6182	2206	696	143	34	5	1	0	0	0	0	0	0	330051
	2	1159	3772	18598	24228	12226	4894	7661	11932	7365	3669	1681	666	175	37	11	1	0	0	0	0	0	98075
	3	10	583	1364	3940	5404	2053	2820	6782	5438	3983	2413	1231	462	188	54	12	2	0	0	0	0	36739
	4	0	15	113	279	778	55	653	2462	2560	2349	1883	1428	768	302	122	33	9	3	0	0	0	13812
**SEWS**	5	0	0	2	19	53	11	44	369	809	1021	1044	1024	763	442	184	76	28	9	1	0	0	5899
	6	0	0	0	0	2	0	3	15	105	227	365	503	485	368	213	112	48	12	3	0	0	2461
	7	0	0	0	0	0	0	0	1	13	27	83	166	158	197	151	89	59	17	8	0	2	971
	8	0	0	0	0	0	0	0	0	3	4	19	30	62	98	64	64	40	22	9	2	1	418
	9	0	0	0	0	0	0	0	0	0	0	3	5	13	19	40	35	22	20	11	3	0	171
	10	0	0	0	0	0	0	0	0	0	0	0	2	2	4	11	10	12	6	9	4	1	61
	11	0	0	0	0	0	0	0	0	0	0	0	0	0	1	1	3	2	2	4	0	0	13
	12	0	0	0	0	0	0	0	0	0	0	0	0	0	0	0	1	3	2	1	1	0	8
	13	0	0	0	0	0	0	0	0	0	0	0	0	0	0	0	0	0	1	0	1	0	2
	Total	295052	344547	219041	115080	52108	20079	19639	39910	23919	13755	8233	5209	2923	1661	852	436	225	94	46	11	4	1,162,824
	Cum Total	294846	639036	857663	992306	1063551			1102881	1126214	1139456	1147276	1152092	1154736	1156173	1156904	1157262	1157439	1157501	1157530	1157539	1157540	

Of the 39178 that NEWS2 = 3 in any single parameter, (1.9%) 766 triggered the lowest SEWS threshold ≥4
Of the 39910 that NEWS2 = 5, 2847 triggered (7.1%) the lowest SEWS threshold ≥4
Of the 23919 that NEWS2 = 6, 3490 triggered (14.5%) the lowest SEWS threshold ≥4
Cum Total Is the cumulative total at or below the NEWS2 score *without an event* used to calculate the odds ratio of the composite outcome event when the Index NEWS2 ≥ threshold score *vs.* < threshold score (Fig S3b).

Table S3b outcome events linked to All NEWS2 and SEWS

		NEWS2																				
		0	1	2	3	4	3 single	4 single	5	6	7	8	9	10	11	12	13	14	15	16	17	18	Total SEWS
SEWS	0	200	208	194	150	73	0	1	37	16	5	0	0	0	0	0	0	0	0	0	0	0	884
	1	3	140	162	163	152	39	29	168	108	56	16	4	0	0	0	0	0	0	0	0	0	1040
	2	3	7	55	94	81	30	63	194	188	117	71	37	14	1	2	0	0	0	0	0	0	957
	3	0	2	3	23	55	15	31	135	159	189	130	80	32	16	7	1	0	0	0	0	0	878
	4	0	0	0	2	5	0	12	40	89	96	108	118	62	47	14	1	1	0	0	0	0	595
	5	0	0	0	0	0	0	0	6	24	40	60	81	88	63	23	13	7	2	0	0	0	407
	6	0	0	0	0	0	0	0	0	2	7	24	50	54	54	23	14	5	3	0	0	0	236
	7	0	0	0	0	0	0	0	0	0	1	3	17	24	31	30	17	11	6	1	0	2	143
	8	0	0	0	0	0	0	0	0	0	2	1	5	3	11	14	19	14	11	4	1	0	85
	9	0	0	0	0	0	0	0	0	0	0	0	0	2	1	4	10	5	6	6	0	0	34
	10	0	0	0	0	0	0	0	0	0	0	0	1	0	0	4	2	3	4	3	1	1	19
	11	0	0	0	0	0	0	0	0	0	0	0	0	0	0	0	0	1	0	3	0	0	4
	12	0	0	0	0	0	0	0	0	0	0	0	0	0	0	0	1	1	0	0	0	0	2
	13	0	0	0	0	0	0	0	0	0	0	0	0	0	0	0	0	0	0	0	0	0	0
	Total	206	357	414	432	366	84	136	580	586	513	413	393	279	224	121	78	48	32	17	2	3	5284
	Cum Total	206	563	977	1493	1995			2575	3161	3674	4087	4480	4759	4983	5104	5182	5230	5262	5279	5281	5284	

Of the 220 that NEWS2 = 3 in any single parameter 12 (5.4%) triggered the lowest SEWS threshold ≥4
Of the 580 that NEWS2 = 5, 46 triggered (7.9%) the lowest SEWS threshold ≥4
Of the 586 that NEWS2 = 6, 115 triggered (19.6%) the lowest SEWS threshold ≥4
5284 outcome events are recorded because every NEWS2 is treated independently and linked to an outcome event over the subsequent 24 hours.
Cum Total Is the cumulative total at or below the NEWS2 score *with an event* used to calculate the odds ratio of the composite outcome event when the Index NEWS2 ≥ threshold score *vs.* < threshold score (Fig S3b).

Supplementary Table S4 bed occupancy by the in-patient population at midday

Group	Number of Beds occupied at 12.00[a]
**Patients eligible for NEWS2 analysis**	
emergency admission in hospital < 24 hours at 12.00	98 ± 15
emergency admission in hospital ≥24 hours at 12.00	632 ± 31
elective admission in hospital <24 hours at 12.00	150 (IQR 53-162)
elective admission in hospital ≥24 hours at 12.00	198 ± 21
**Patients not eligible for NEWS2 analysis**	
ICU	72 (IQR: 67-76)
DNACPR	170 ± 15

Supplementary Table 4 legend

Number of beds occupied at midday for the 273 days between 01/11/2018 and 31/7/2019.

[a] Mean ± standard deviation for normally distributed groups. Median and interquartile range if not normally distributed. (Elective admission in hospital <24 hours included day case admissions which vary significantly with the day of the week). In those eligible for NEWS2 analysis, weekday bed occupancy = 1096 (median; IQR: 1079-1113) and weekend bed occupancy = 949 (median; IQR: 937-976). Across all days, the mean daily bed occupancy at midday in those eligible for NEWS2 analysis = 1046.
