# Supplementary material for: Evaluation of NEWS2 response thresholds in a retrospective observational study from a UK acute hospital
Source: BMJ Open. 2022 Feb 8;12(2):e054027. doi: 10.1136/bmjopen-2021-054027 (PMC8830252; doi:10.1136/bmjopen-2021-054027)
Supplement: Author's manuscript [file bmjopen-2021-054027.draft_revisions.pdf]

BMJ Open is committed to open peer review. As part of this commitment we make the peer review history of every article we publish publicly available.

When an article is published we post the peer reviewers' comments and the authors' responses online. We also post the versions of the paper that were used during peer review. These are the versions that the peer review comments apply to.

The versions of the paper that follow are the versions that were submitted during the peer review process. They are not the versions of record or the final published versions. They should not be cited or distributed as the published version of this manuscript.

BMJ Open is an open access journal and the full, final, typeset and author-corrected version of record of the manuscript is available on our site with no access controls, subscription charges or pay-per-view fees (http://bmjopen.bmj.com).

If you have any questions on BMJ Open's open peer review process please email info.bmjopen@bmj.com

BMJ Open

Are current NEWS2 clinical response thresholds optimised for a general in-patient population?

Journal:	*BMJ Open*
Manuscript ID	bmjopen-2021-054027
Article Type:	Original research
Date Submitted by the Author:	02-Jun-2021

Complete List of Authors:	Pankhurst, Tanya ; University Hospitals Birmingham NHS Foundation Trust,<br>Sapey, Elizabeth; University of Birmingham, Institute of Inflammation and Ageing<br>Gyves, Helen; University Hospitals Birmingham NHS Foundation Trust, Health Informatics<br>Evison, Felicity; University Hospitals Birmingham NHS Foundation Trust, Department of Health Informatics<br>Gallier, Suzy; University Hospitals Birmingham NHS Foundation Trust, Health Informatics; University of Birmingham, PIONEER Technical Director<br>Gkoutos, George; University of Birmingham,<br>Ball, Simon; University Hospitals Birmingham NHS Foundation Trust; Health Data Research UK, Better Care

Keywords:	INTERNAL MEDICINE, Health policy < HEALTH SERVICES ADMINISTRATION & MANAGEMENT, Health informatics < BIOTECHNOLOGY & BIOINFORMATICS

SCHOLARONE™
Manuscripts

I, the Submitting Author has the right to grant and does grant on behalf of all authors of the Work (as defined in the below author licence), an exclusive licence and/or a non-exclusive licence for contributions from authors who are: i) UK Crown employees; ii) where BMJ has agreed a CC-BY licence shall apply, and/or iii) in accordance with the terms applicable for US Federal Government officers or employees acting as part of their official duties; on a worldwide, perpetual, irrevocable, royalty-free basis to BMJ Publishing Group Ltd ("BMJ") its licensees and where the relevant Journal is co-owned by BMJ to the co-owners of the Journal, to publish the Work in this journal and any other BMJ products and to exploit all rights, as set out in our licence.

The Submitting Author accepts and understands that any supply made under these terms is made by BMJ to the Submitting Author unless you are acting as an employee on behalf of your employer or a postgraduate student of an affiliated institution which is paying any applicable article publishing charge ("APC") for Open Access articles. Where the Submitting Author wishes to make the Work available on an Open Access basis (and intends to pay the relevant APC), the terms of reuse of such Open Access shall be governed by a Creative Commons licence – details of these licences and which Creative Commons licence will apply to this Work are set out in our licence referred to above.

Other than as permitted in any relevant BMJ Author's Self Archiving Policies, I confirm this Work has not been accepted for publication elsewhere, is not being considered for publication elsewhere and does not duplicate material already published. I confirm all authors consent to publication of this Work and authorise the granting of this licence.

For peer review only

Are current NEWS2 clinical response thresholds optimised for a general in-patient population?

Dr Tanya Pankhurst[1], Dr Elizabeth Sapey[2], Ms Helen Gyves[3], Ms Felicity Evison[4], Ms Suzy Gallier[5], Professor Georgios Gkoutos[6], Professor Simon Ball[1, 7]

Affiliations

1. University Hospitals Birmingham NHS Foundation Trust, Edgbaston, Birmingham, UK Tanya.Pankhurst@uhb.nhs.uk

2. a. PIONEER, HDRUK Health Data Hub in Acute Care, Birmingham, UK. University of Birmingham, Edgbaston, Birmingham, UK, B15 2GW. Institute of Inflammation and Ageing, University of Birmingham, Birmingham, UK. ORCiD ID: 0000-0003-3454-5482. Email: e.sapey@bham.ac.uk Tel: 00 44 121 246 2000

b. Acute Medicine, University Hospitals Birmingham NHS Foundation Trust, Edgbaston, Birmingham, UK.

3. University Hospitals Birmingham NHS Foundation Trust, Edgbaston, Birmingham, UK. Helen.Gyves@UHB.nhs.uk

4. University Hospitals Birmingham NHS Foundation Trust, Edgbaston, Birmingham, UK. Felicity.Evison@UHB.nhs.uk

5. University Hospitals Birmingham NHS Foundation Trust, Edgbaston, Birmingham, UK. Suzy.Gallier@UHB.nhs.uk

6. Health Data Research UK (HDR-UK) Midland's Physical Site, Birmingham, UK. Institute of Cancer and Genomic Sciences, University of Birmingham, Birmingham, UK. G.Gkoutos@bham.ac.uk

7. Health Data Research UK (HDR-UK) Midland's Physical Site. University of Birmingham, Edgbaston, Birmingham, UK. Institute of Immunology and Immunotherapy, University of Birmingham, Birmingham, UK. simon.ball@uhb.nhs.uk

Corresponding author: Professor Elizabeth Sapey. e.sapey@bham.ac.uk

For peer review only - http://bmjopen.bmj.com/site/about/guidelines.xhtml

Key words

Abstract word count: 299

Main article word count: 3980 (excluding citations)

References: 38

Tables: 3

Figures: 2

There is an online supplement

For peer review only

For peer review only - http://bmjopen.bmj.com/site/about/guidelines.xhtml

ABSTRACT

Objective: Use of the National Early Warning Score2 (NEWS2) has been mandated in adults admitted to acute hospitals in England. Urgent clinical review is recommended at NEWS2≥5. This policy is recognised as requiring ongoing evaluation. We assessed NEWS2 acquisition, alerting at key thresholds, and patient outcomes, to understand how response recommendations affect clinical resource allocation.

Setting: Adult acute hospital in England.

Design: Observational cohort study.

Participants: 100,362 consecutive admissions between Nov 2018-Jul 2019.

Outcome: Death or admission to ICU within 24 hours of a score.

Methods: NEWS2 were assembled as a single score from consecutive 24-hour timeframes; the first score recorded ("Index-NEWS2"), the highest NEWS2, or as all scores from the admission ("All-NEWS2"). Scores were excluded from analysis when a patient was in intensive care, in the presence of a decision not to undertake cardiopulmonary resuscitation, or on Day 1 of elective admission prior to the planned procedure.

Results: A mean of 4.5 NEWS2 were acquired per patient per day. The outcome rate following an Index-NEWS2 was 0.22/100 patient-days. The sensitivity of outcome prediction at Index-NEWS2 ≥5 = 0.46, and number needed to evaluate (NNE) =52. At this threshold, a mean of 37.6 alerts/100 patient-days would be generated, occurring in 12.3% of patients on any single day. Threshold changes to increase sensitivity by 0.1, would result in a 2-fold increase in alert rate and 1.5-fold increase in NNE. Overall, NEWS2 classification performance was significantly worse on Index-scores than All-scores (c-statistic = 0.78 vs 0.85; p<0.001).

Conclusions: The combination of low event-rate, high alert-rate and low sensitivity, in patients for cardiopulmonary resuscitation, means that at current NEWS2 thresholds, resource demand is significant, and might redirect care from other patients at need. In analyses that epitomise in-patient screening, NEWS2 performance suggests a need for re-evaluation of current response recommendations in this population.

For peer review only

Strengths and limitations of the study

- All admissions to an acute hospital within the study timeframe are included, providing the basis for a detailed understanding of the consequences of NEWS2 based policy.

- A precise definition of DNACPR decisions over the course of an admission informed our analysis, in order to maintain correspondence with decision making and treatment options available clinically.

- The evaluation of NEWS2 as a single score from discrete 24-hour timeframes, provides an assessment of classification performance which epitomises its role in screening patients between routine reviews, moderating the effects of including large numbers of scores acquired from those already identified to be at clinical risk.

- The analysis is limited by the fact that this is a single centre study, however the underlying data are consistent with other reports from acute hospitals in the NHS.

- The analysis excludes Day 1 of elective admission from outcome-based analysis, because deranged physiology was predicted to occur following planned intervention on that day.

For peer review only

For peer review only - http://bmjopen.bmj.com/site/about/guidelines.xhtml

INTRODUCTION

The use of early warning scores (EWS's) has been widely advocated, to integrate physiological parameters into a single actionable output. In 2017, the Royal College of Physicians (RCP) published a modified National Early Warning Score (NEWS), referred to as NEWS2 [1]. NEWS2 is a scoring system based upon six physiological parameters. It is associated with specific clinical response recommendations, including urgent clinical review at a key threshold NEWS2 score ≥5. This requires attendance by clinicians with competence in the assessment and treatment of acutely ill patients, and where necessary, escalation to a team with critical care competencies [1]. At NEWS2 score ≥7, this is uplifted to assessment by a team with those critical care competencies. In 2019, the National Health Service in England (NHSE) mandated the use of NEWS2 for all adults in acute hospitals and widened its application to include screening for sepsis [2]. This extends its use significantly beyond the evaluation of acute admissions, for which NEWS was initially developed and validated [3-8]. The National Institute for Health and Care Excellence (NICE) has identified the need for further evaluation of NEWS2, to ensure no adverse consequences from its roll out across the NHS [9]. This is in the context of limited evidence of survival benefit from an EWS triggered response [8, 10-15], or definition of the resource required to deliver a response [16]. Since electronic systems directly link a threshold EWS to a response recommendation [17], high rates of alerting may arise, unmoderated by human intervention [18, 19], creating an opportunity for clinical resource to be diverted in ways that could be counterproductive [9].

Given a requirement to significantly alter clinical practice [1, 2], we evaluated the performance of NEWS2, across the in-patient population of an acute hospital. This included a description of the rate of NEWS2 data acquisition, the rates at which key threshold scores were met, and their relationship to outcome. This was with a view to understanding how NEWS2 based recommendations could affect the distribution of resource when put into practice, taking into account factors which may modify the outcome, such as resuscitation status, or those which may amplify demand, such as recurrent alerting [9].

METHODS

Setting

The Queen Elizabeth Hospital Birmingham (QEHB) is a National Health Service (NHS), urban, adult, acute hospital in England with 1269 beds including 80 level 2/3 intensive care (ICU) beds, an Emergency Department that assesses >300 patients per day, and a mixed secondary and tertiary practice that includes all major adult specialities with the exception of obstetrics and gynaecology. The EHR at QEHB (PICS, Birmingham Systems) contains time-stamped, structured records that include demography, location, time of admission and discharge, physiological measurements supporting NEWS2 and Standard Early Warning Score (SEWS) (Table S1 of the online supplement) and Do Not Attempt Cardiopulmonary Resuscitation (DNACPR) decisions.

NEWS2 data collection was electronically mandated, whilst alerting continued to use established SEWS thresholds [20], (coincidentally facilitating an assessment of NEWS2 performance at the key action threshold ≥5, minimally disrupted by triggered clinical responses (Table S2 and S3 of the online supplement)). Existing standards included a maximum interval between EWS acquisition = 12 hours, progressive alerting to ward-based staff and automated escalation to a 24/7 critical care outreach team at threshold SEWS.

Cohort and Definitions

All hospital spells (continuous stay in a hospital bed), between 00.00 on 01/11/2018 and 23.59 on 31/7/2019, were evaluated to discharge (99.6%), or to 56 days post admission (0.4%) if that was earlier. Initial Emergency Department assessments, prior to admission, were not included. An adapted consort diagram is shown in Figure 1a. Admissions were identified as emergency or elective from the mandatory provider spell admission method code. A composite outcome event was defined as the first of unplanned admission to ICU (type 1 and 2 of the NHS critical care minimum dataset) or death of the patient, within 24 hours of a NEWS2 score.

In the primary analysis, each spell was divided into consecutive 24-hour post-admission days starting at the time of admission. As shown in Figure 1b, for admission time T, consecutive 24-hour periods end at T + (n x 24 hours); where n is the nominal post-admission day. The first NEWS2 recorded in each post-admission day, at time t_n was termed the Index-NEWS2 score. An overlapping, patient-day variable was defined between t_n and t_n+24hours. The first outcome occurring in the patient-day was linked to the preceding Index-NEWS2. This design was used to

ensure a single NEWS2 score was captured from discrete, consecutive, 24-hour time periods and that the outcome was assessed over 24 hours.

In addition to Index-NEWS2, analyses were undertaken using all the scores recorded in a given time period (termed All-NEWS2) [21] and using the highest score on each post-admission day (termed Highest-NEWS2). NEWS2 scores were not eligible for inclusion if at the time the score was acquired, the patient was in ICU or had a DNACPR in place. This was to achieve internal consistency when using admission to ICU as part of the composite outcome, since decisions not to resuscitate are highly concordant with ceiling of care not including ICU. On return from ICU or if a DNACPR was revoked, subsequent scores were included in the analysis. No score was calculated when patients underwent an operative procedure away from the ward.

Day 1 post elective-admission was excluded from outcome-based analysis because it was known that almost all were admitted for surgery later that day, after a NEWS2 was recorded. Any relationship with outcome might then be confounded by decisions not to proceed to intervention on that day, informed by NEWS2 or its component observations.

Patient demographics are reported for the first admission in the study period. Additional context is provided by reporting bed occupancy at midday across the study period (Table S4 of the online supplement).

Analysis of EWS performance

To evaluate the performance of different NEWS2 threshold scores, a patient with a NEWS2 ≥ threshold score was defined as predicted positive (P) and a patient with a NEWS2 < threshold score as predicted negative (N). At different NEWS2 threshold values, performance metrics were calculated from 4 different groups: true positive (TP), when an outcome event was correctly predicted, false positive (FP) when an outcome event was predicted but did not occur; with true negative (TN) and false negative (FN) following suit. Receiver operating characteristics (ROC's) were derived from these metrics, for Index-NEWS2 and All-NEWS2.

A range of performance metrics were calculated including two identified to be particularly suited to the representation of early warning score performance, namely the Number Needed to Evaluate

(NNE) and the Alert Rate [22]. NNE is the number of patients meeting a threshold NEWS2 score, to include one who then sustains an outcome event, defined as follows:

$$NNE = \frac{TP + FP}{TP} = \frac{1}{PPV}$$

The Alert Rate is the number of threshold scores (alerts that would be generated) per 100 inpatients per day, defined as follows:

$$Alert\ Rate = \frac{TP + FP}{TP + FP + TN + FN}\ x\ number\ of\ NEWS2\ scores\ acquired\ (/100\ patients/day)$$

The NNE and Alert Rate were plotted against the sensitivity as previously discussed [22]. For the Index-NEWS2, an outcome event rate per 100 patient-days, was calculated (Overall Event Rate) as follows:

$$Overall\ Outcome\ Event\ Rate = \frac{TP + FN}{TP + FP + TN + FN}\ x\ 100\ /100\ \text{patient days}$$

For the Index-NEWS2, an outcome event rate at any given threshold score (Alerted Event Rate) per 100 patient-days, was calculated as follows:

$$Alerted\ Outcome\ Event\ Rate = \frac{TP}{TP + FP + TN + FN}\ x\ 100\ /100\ \text{patient days}$$

For the purpose of reporting any daily rate, time of discharge was not considered, a whole day was counted.

Estimation of Clinician Resource Requirement

The clinical resource required to support response recommendations was illustrated by assuming 1 hour of healthcare professional time per clinical evaluation, informed by a report of a rapid clinical response team on a surgical ward in Holland [23]. The number of healthcare professionals required /100 beds occupied was calculated based upon a 40-hour working week and 18% overhead for leave (=1760 hours/year).

For peer review only - http://bmjopen.bmj.com/site/about/guidelines.xhtml

Statistical analysis

All statistical analyses were undertaken in STATA SE 15.1. Normally distributed variables are represented as mean +/- standard deviation others as median and interquartile range. Bootstrap analyses of the ROC were undertaken with 10,000 repetitions of 10,000 patient-days. There were no adjustments for multiple comparisons and all p values are reported.

Patient and public involvement

302 patients and public members were consulted in relation to the use of health data to improve the care for people with acute, unplanned illness. The theme of acuity scores was discussed in a working group and agreed to be a priority for patients. Access to health data was specifically discussed with a group of patients and members of the public, who reviewed data field names and agreed data access was in the interests of patients. Initial results have been fed back to patient groups. The full results of this paper will be disseminated through the PIONEER patient and public group.

Research Ethics Approvals

This study was approved by the East Midlands–Derby REC (reference: 20/EM/0158) and as part of a change assessment programme (UHB clinical audit: CARMS-15850).

For peer review only - http://bmjopen.bmj.com/site/about/guidelines.xhtml

RESULTS

Patients and admissions

There were 100,362 admissions across 273 days. In 21,744 no NEWS2 was recorded, 21,599 (99.3%) of which were associated with a short length of stay < 12 hours (Figure 1a). The other 78,431 admissions in which NEWS2 was recorded, occurred in 52,214 patients (Table 1).

DNACPR and outcome

A DNACPR decision was made in 4,621 (4.6%) admissions. This resulted in a DNACPR that was active in 170 ± 15 (13%) in-patients at midday (Table S4 of the online supplement). Of 1076 deaths not on ICU, 943 (87.6%) occurred in those with a DNACPR decision, in 834 of whom this had been in place >24 hours.

Index-NEWS2 recording and outcome

A DNACPR was active throughout the course of 367 admissions. In the remaining 77,877 admissions, 294,602 Index-NEWS2 were recorded. They were associated with 715 outcome events in the following 24 hours (154 deaths and 561 admissions to ICU). Of 10,731 post-admission days in which no NEWS2 was calculated, 5,786 (53.9%) involved discharge later that day, whilst 4,424 (41.2%) comprised a whole day in which the patient was eligible for inclusion and in which no outcome event occurred (Figure S1 of the online supplement).

Day 1 post-elective admission

Exclusion of Day 1 of elective admission from the analysis of Index-NEWS2 associated outcome, was supported by finding that 94.7% of admissions were followed by a planned procedure, that this occurred within 12 hours, and all outcome events followed a procedure.

Classification performance of NEWS2

Across all other days, 1,162,824 All-NEWS2 scores were recorded on 258,678 post-admission days, so a mean of 4.5 NEWS2 were recorded per day. 580/258,678 (0.22%) Index-NEWS2 were associated with an outcome event. 5,284/1,162,824 (0.45%) All-NEWS2 were associated with an outcome event, since a mean of 9 NEWS2 were recorded in the 24 hours prior to an outcome event. The c-

For peer review only - http://bmjopen.bmj.com/site/about/guidelines.xhtml

statistic of outcome prediction was higher when derived from All-NEWS2 compared to Index-NEWS2 (0.85 vs 0.78; p<0.001; Figure S2 of the online supplement).

Alert Rate and NNE associated with Index-NEWS2

Table 2 presents Index-NEWS2 performance. The Index-NEWS2 Alert Rate is shown in Table 3. Figure 2a plots this Alert Rate against sensitivity for the Index-NEWS2 score. Around current key clinical response thresholds, the relationship was log-linear: a 2.0 x increase in the Alert Rate for an increase in sensitivity of 0.1 (Alert Rate = $0.19e^{7.18\text{xsensitivity}}$ between NEWS2 ≥10 and ≥2). At the key action threshold NEWS2 ≥5 the Alert Rate generated by an Index-NEWS2 = 5.3/100 patients/day, at which score the Index-NEWS2 sensitivity = 0.46.

Figure 2b shows the relationship between the NNE and sensitivity. Around current key clinical response thresholds, the relationship was log-linear: a 1.5 x increase in NNE to increase sensitivity by 0.1 (NNE = 8.7 $e^{4.14\text{xsensitivity}}$ between NEWS2 ≥10 and ≥2). At the key action threshold NEWS2 score ≥5, the NNE = 52.

Alert Rate associated with All-NEWS2

The Alert Rate at a threshold Index-NEWS2 score represents the rate derived from that single score. However, a mean of 4.5 NEWS2 scores were recorded per post-admission day. The Alert Rate derived from All-NEWS2 scores is shown in Table 3. Figure 2a plots the Alert Rate generated by All-NEWS2 against the sensitivity of Index-NEWS2, across different thresholds. Around current clinical response thresholds, the relationship was log-linear: a 1.8 x increase in the Alert Rate for an increase in sensitivity of 0.1 (Alert Rate = $2.12e^{6.14\text{xsensitivity}}$ between NEWS2 ≥10 and ≥2). At the key action threshold NEWS2 score ≥5, the Alert Rate generated by All-NEWS2 = 37.6/100 patients/day.

Alert Rate associated with Highest-NEWS2

The Alert Rate generated by the Highest NEWS 2 score in each post-admission day is also shown in Table 3. Figure 2a plots the Alert Rate generated by the Highest-NEWS2, against the sensitivity of the Index-NEWS2, across different thresholds. Around the current clinical response thresholds, the relationship was log-linear: a 1.8 x increase in the Alert Rate for an increase in sensitivity of 0.1 (Alert Rate = $0.73e^{6.07\text{xsensitivity}}$ between NEWS2 ≥10 and ≥2). At the key action threshold NEWS2 score ≥5, the Alert Rate generated by the Highest-NEWS2 = 12.3/100 patients/ day.

For peer review only - http://bmjopen.bmj.com/site/about/guidelines.xhtml

Incremental clinical resource required by changes to NEWS2 threshold

The number of clinicians required to support responses deployed at the different NEWS2 thresholds, is also estimated in Table 3. This is derived from the Alert Rates defined by the Index-NEWS2, All-NEWS2 and the Highest-NEWS2 at each threshold, with 1 hour assigned per clinical response deployed. Thus, at the key action threshold NEWS2 score ≥5, demand for clinician resource was calculated to be respectively 1.1, 7.8 and 2.6 WTE clinicians/100 in-patients. This would equate to 14, 98 and 32 WTE clinical staff to respond to the Index, All or the highest NEWS2 respectively in the QEH (1269 beds).

Calibration of NEWS2

Although not developed as a predictor of absolute risk, NEWS2 usefully represents progression of risk across the population analysed. Integer changes in NEWS2 threshold were associated with approximately equal relative changes in the odds ratio of outcome events, across a wide range of scores (Figure S3 of the online supplement).

For peer review only

For peer review only - http://bmjopen.bmj.com/site/about/guidelines.xhtml

DISCUSSION

In 2019, NHSE required that NEWS2 be used to monitor all adults in acute hospitals [2], extending its scope to include screening for sepsis. This was linked to clinical response recommendations defined by the RCP, including a key threshold for urgent clinical review at NEWS2≥5 [1]. NICE cautioned that when used in this way, there was a risk that NEWS2 based recommendations could result in new demand, which paradoxically might adversely affect overall care delivery [9]. A need for ongoing evaluation was identified. Our analysis was designed to provide a description of how NEWS2 implementation could affect the disposition of clinical resource, across an acute hospital in-patient population, considering the effects of response modifiers such as resuscitation status as well as repeated measurement in those already identified for increased levels of care.

In contrast to other studies, NEWS2 recorded at the time of a DNACPR was excluded from our analysis. This was possible because time stamped records of both were available in the EHR. Previous reports have excluded patients on end-of-life care pathways, however this had to be inferred from an absence of observations in the 24 hours prior to death [24-26]. Precise delineation of these two groups shows that the majority of deaths occurred in patients with a DNACPR in place for more than 24 hours, whereas ICU admissions occurred in patients without a DNACPR. Although this may be unsurprising, it is important when considering the translation of NEWS2 into clinical practice. Compared to previous reports [24-26], there was a low event rate in the large group of patients who were eligible for outcome-based analysis, because the majority of deaths occurred in patients with a DNACPR. A low event rate in this group is one reason why NEWS2 may not perform as well as expected when translated into clinical practice [9, 15]. It is evident in metrics sensitive to the event rate, such as the NNE. This analysis does not imply that NEWS2 is not applicable to patients with a DNACPR, but it does reflect difficulty in interpreting the real-world consequences of recommended thresholds, if these populations are not analysed separately.

Index-NEWS2 was used to assemble single scores from discrete post-admission timeframes, to limit over-representation of scores recorded for clinical indication rather than routine screening. Clinical practice guidelines, including those associated with NEWS2 [1], require increased monitoring of physiological parameters to track those already identified to be at risk of further deterioration. This requires a progressive representation of risk, a different task to screening a population using routine observations. Our findings are relevant to the latter. The better classification performance of NEWS2

on All-scores compared to Index-scores implies that NEWS2 discriminates better on days in which data acquisition is more frequent. This conclusion is apparently different to the influential report of Jarvis and colleagues, which found that the c-statistic of NEWS and other scoring systems are little affected when using all or single scores per admission [21]. However, their random selection of single scores across an admission would be expected to mirror the distribution of all scores. In contrast, a methodology employing single scores from a timeframe typical of that between routine reviews [27, 28] is less influenced by superior performance on scores obtained during times of high frequency monitoring. Arguably, our analysis of Index-NEWS2 more closely reflects its use as a screening tool in the interval between ward-round based routine assessment, which is typically 24 hours. This difference in performance is another reason why in clinical practice, NEWS2 might not perform as well as expected from the literature [15].

Even at the lower action threshold ≥5, Index-NEWS2 sensitivity was less than 0.5. Other routes to clinical evaluation, such as routine or symptom-based assessment are therefore also likely to play an important role in identifying deteriorating patients for assessment. The resource with which to deliver these assessments must then compete with that demanded by NEWS2 triggered responses. Diversion of resource is one mechanism by which NEWS2 implementation could adversely affect patient care, particularly if alert rates are high [9].

In our centre, alert rates would be high at current NEWS2 thresholds. This is consistent with data from other NHS acute hospitals [24, 26]. A meaningful diversion of clinical resource away from other routes to evaluation is therefore possible [9]. Equally, alert fatigue could arise, thereby degrading clinical decision making [29]. In response to this, some centres have developed local policies to manage repeated alerting in some situations [30].

Given that responses to All-NEWS2 may be modified in various ways, including prospective decision-making or censoring by ward-staff at the point of care [31], we also defined an Alert Rate based on the Highest-NEWS2 score each day. This is operationally equivalent to allowing one triggered response per person per day. Although it might underestimate the optimal response rate, this provides additional context with which to understand the boundaries of reasonable clinical resource deployment.

For peer review only - http://bmjopen.bmj.com/site/about/guidelines.xhtml

An optimal threshold score would usually be defined by health economic analysis [8, 32]. This is not possible because there is little evidence quantifying survival benefit from EWS triggered responses [8, 10-16], nor is there a consistent account of the cost of current clinical response recommendations. We assigned 1 hour of clinician time per response, to illustrate the potential resource implications of different thresholds. This was based on a single informative study [23] identified by NICE guidance on emergency and acute care [16]. It evaluated a team with critical care competencies, most like that indicated for NEWS2≥7. We chose not to vary this resource attribution further, as our aim was to provide perspective on scale rather than precise definition of cost.

Although a health economic analysis is not possible, description of the Alert Rate and Event Rate provides some insight into the resource consequences of different NEWS2 thresholds, across an acute hospital in-patient population. For example, reducing the NEWS2 threshold from ≥7 to ≥5 would increase the Alert Rate from 4.1 to 12.3 /100 post-admission days, thereby increasing the modelled demand for healthcare professionals from 0.9 to 2.6 whole time equivalents /100 in-patients. This resource estimate is based upon Highest-NEWS2, so may be an under-estimate. The number of Index-NEWS2 triggered responses followed by an event would increase by 0.04/100 patient-days, but 0.12 events /100 patient-days would still not have been predicted. Even as an approximation, these results reveal that small changes in current thresholds can result in demand for clinical resource that could meaningfully impact delivery through other care pathways.

Our analysis of NEWS2 excluded patients when there was a DNACPR decision. This is a diverse group of patients, including those approaching the end of life as well as those in whom significant intervention is considered. Since they form a minority of in-patients, their exclusion from outcome-based evaluation would not alter our conclusions regarding the use of undifferentiated NEWS2 thresholds. Nevertheless, this population warrants separate analysis, in particular those whose resuscitation status changed in the 24 hours prior to death. This may inform an understanding of how different implementations of EWS's are associated with different in-hospital cardiac arrest rates, possibly because they prompt DNACPR decisions differently [33].

Our analysis also excluded Day 1 of elective admission. This is because different cause-effect relationships during that day significantly confound the relationship between physiological derangement and outcome. Previous studies have excluded similar cohorts by not including elective

admissions [24-26], or day-case admissions [9, 15]. Again, this exclusion does not imply that NEWS2 is not of use, but that a different outcome analysis would be required, arguably one beginning on return to a ward post-procedure.

A single centre analysis has limitations with respect to generalisation to other populations. Our underlying data are though consistent with reports from other NHS acute hospitals [24, 26]. Furthermore, an editorial response from 2012, employing unpublished data from users of the VitalPac EWS, suggested that RCP recommendations on NEWS (and now NEWS2) would be unsustainable across an entire in-patient population [34]. This can be understood not simply as an issue of capacity but potential jeopardy arising from the redirection of resource [9]. In an acute admissions unit, where event rates are high and resources already targeted, value may be realised from a representation of risk that supports healthcare professionals tracking of patients [3]. As already discussed, this is a different task to the efficient discrimination required of a screening test, applied across the in-patient population, to trigger further clinical evaluation. This distinction is also relevant to recommendations on the identification of sepsis, in which a NEWS2 ≥5 is used to prompt clinical assessment by a senior decision maker [2, 35]. This threshold was developed from the association between outcome and NEWS [36] or qSOFA [24, 25], in those with features of sepsis. NEWS2 ≥5 used as a screening tool for sepsis has not been directly assessed. Our analysis illustrates the potential real-world consequences of such recommendations, including on the distribution of senior clinical resource [9].

In summary, we identify why NEWS2 may not perform as well as expected when screening the in-patient population. This relates not just to moderate classification performance of NEWS2 in this setting (23), but the consequences of high alert rates, including competition for clinical resource [9] and clinicians' attention [29]. There is a particular need to manage multiple alerts in rapid succession [30]. These problems relate in part to the fact that NEWS2 was evolved for paper-based, or stand-alone implementation. In these settings, alert censoring by ward staff is well documented, whether appropriate or not [18, 19]. EHR's automate alerting, thereby generating different problems, associated with high alert-rates. The EHR offers the opportunity to develop more sophisticated scoring systems incorporating a wide range of data, however correspondence with a national, paper-based system would then be lost. Any such development would require careful evaluation using a suitable methodology, such as cluster randomisation [37]. This approach has recently demonstrated improved 30-day mortality in patients identified to be at risk of

For peer review only - http://bmjopen.bmj.com/site/about/guidelines.xhtml

deterioration in Kaiser-Permanente hospitals. An algorithm was used to assess co-morbid conditions and laboratory parameters, as well as physiological parameters [38]. At the chosen response threshold, only 2.8 alerts per 100 patients per day were generated. This was followed by a complex intervention involving remote review by specially trained nurses, stipulated to avoid alert fatigue in hospital staff. It is therefore a significantly different implementation to NEWS2 in England, but one that demonstrates the potential for targeted assessment using an EHR. Indeed, it may be that simple scoring systems are limited in their capacity to confer net benefit across a diverse population, receiving current standards of routine review [27]. Failure to show survival benefit from EWS triggered responses would in that case, not simply reflect limitations in the methodologies used to undertake assessment [15].

In conclusion, there is a risk that currently constituted NEWS2 based response recommendations could adversely impact the overall delivery of care to an in-patient population [9]. The response to multiple alerts requires better definition and ongoing evaluation. As a result, we would not support undifferentiated implementation of current recommendations at a key threshold NEWS2 score ≥5, across the entire in-patient population at our centre. Given existing reports of NEWS2 performance, our findings are likely to be relevant to other acute hospitals.

For peer review only - http://bmjopen.bmj.com/site/about/guidelines.xhtml

TABLES

Table 1 Demographic and clinical characteristics of patients

	Elective	Emergency
Number of patients with one or more included admission eligible for analysis (first admission)	22,538	29,122
Mean age / years (at time of first admission)	53.9 ± 18.1	56.2 ± 21.7
Number of male patients (percentage)	12,142 (53.9%)	13,800 (47.4%)
Ethnicity		
White	16,153 (71.7%)	19,596 (67.3%)
South Asian	2,642 (11.7%)	4,203 (14.4%)
Black	943 (4.2%)	1,489 (5.1%)
Other	806 (3.6%)	1434 (4.9%)
Not Known	1,994 (8.8%)	2,400 (8.2%)
**Admitting speciality on first admission**		
General Medicine	1,545(6.9%)	20,174 (69.3%)
General Surgery	2,150 (9.5%)	3,061 (10.5%)
Trauma & Orthopaedics	2,307 (10.2%)	1,486 (5.1%)
Neurosurgery	1,866 (8.3%)	639 (2.2%)
Urology	1,411 (6.3%)	587 (2.0%)
Cardiology	1,821 (8.1%)	469 (1.6%)
Clinical & Medical Oncology	1,263 (5.6%)	549 (1.9%)
Ear, Nose and Throat (ENT)	1,401 (6.2%)	389 (1.3%)
Plastic Surgery	2,138 (9.5%)	304 (1.0%)
Maxillo-Facial Surgery	849 (3.8%)	252 (0.9%)
All others	5,787 (25.7%)	1,212 (4.2%)
Mean number of admissions / patient	1.6	1.5
Number of admissions in which the patient was ineligible for analysis throughout an admission	60	494

Table 1 Legend

The demographics, mode of admission and admitting speciality on first admission, of the 52,214 patients that were subject to NEWS2 analysis, contributing 36,182 elective and 42,249 emergency admissions. In 554 admissions the patient was ineligible for inclusion in analysis throughout the spell (187 due to admission and discharge from ICU. 367 due to a DNACPR decision).

For peer review only

For peer review only - http://bmjopen.bmj.com/site/about/guidelines.xhtml

Table 2 Performance of Index NEWS 2

Threshold NEWS2	Number of Index NEWS2 meeting threshold	Number of composite outcome events associated with Index NEWS2 at threshold	Alert Rate / 100 patients / post-admission day	Sensitivity (%)	Specificity (%)	PPV (%)	NPV (%)	NNE	Alerted outcome event rate / 100 patient-days
**≥1**	184,867	534	71.5	92.1	28.6	0.3	99.9	347	0.21
**≥2**	101,364	453	39.2	78.1	60.9	0.4	99.9	224	0.17
**≥3**	53,713	383	20.8	66.0	79.3	0.7	99.9	141	0.15
**≥4**	26,484	318	10.4	54.7	89.9	1.2	99.9	84	0.12
**≥5 + Single=3**	21,833	288	8.4	49.6	91.7	1.3	99.9	76	0.11
**≥5**	13,793	268	5.3	46.1	94.8	1.9	99.9	52	0.10
**≥6**	7,252	216	2.8	37.1	97.3	3.0	99.9	34	0.08
**≥7**	3,824	153	1.5	26.2	98.6	4.0	99.8	25	0.06
**≥8**	2,095	105	0.8	18.0	99.2	5.0	99.8	20	0.04
**≥9**	1,158	75	0.4	12.8	99.6	6.4	99.8	16	0.03
**≥10**	613	51	0.2	8.6	99.8	8.2	99.8	12	0.02

Table 2 legend

The Index NEWS2 score is the first recorded in each consecutive 24-hour period post-admission. 258,678 Index NEWS2 score were analysed from Day 1 to Day 56 of admission, excluding the first day of elective admission. There were 580 outcome events.

The alerted outcome event rate per 100 patient days is the number of outcome events following an Index NEWS2 meeting the threshold NEWS2 score. The overall outcome event rate = 0.22 per 100 patient-days. PPV is positive predictive value. NPV is negative predictive value. NNE is number needed to evaluate.

For peer review only

For peer review only - http://bmjopen.bmj.com/site/about/guidelines.xhtml

Table 3
Alert Rates and clinician resource required to respond at threshold NEWS2

Threshold NEWS2	Index NEWS2 Alert Rate / 100 patients / post-admission day	WTE clinician / 100 occupied beds to respond to Index NEWS2 at threshold	All-NEWS2 Alert Rate / 100 patients / post-admission day	WTE clinician / 100 occupied beds to respond to All NEWS2 at threshold	Highest-NEWS2 Alert Rate / 100 patients / post-admission day	WTE clinician / 100 occupied beds to respond to Highest NEWS2 at threshold
≥1	71.5	14.8	335.4	69.6	91.4	19.0
≥2	39.2	8.1	202.3	41.9	64.5	13.4
≥3	20.8	4.3	117.6	24.4	39.6	8.2
≥4	10.4	2.2	65.3	13.6	22.1	4.6
≥5 or Single=3	8.4	1.7	52.9	11.0	19.3	4.0
≥5	5.3	1.1	37.6	7.8	12.3	2.6
≥6	2.8	0.6	22.2	4.6	7.1	1.5
≥7	1.5	0.3	12.9	2.7	4.1	0.9
≥8	0.8	0.2	7.6	1.6	2.5	0.5
≥9	0.4	0.1	4.4	0.9	1.5	0.3
≥10	0.2	<0.1	2.4	0.5	0.9	0.2

Table 3 legend

The Alert Rate is the number of NEWS2 scores that reach threshold per 100 patients per post-admission day. NEWS2 score were analysed from Day 1 to Day 56 of admission, excluding the first day of elective admission. Index NEWS2 reports the first NEWS2 recorded in each post-admission day. All-NEWS2 includes all scores recorded in the admission spell. Highest-NEWS2 reports the highest score recorded in each post-admission day. The whole time equivalent (WTE) clinician resource required to service clinical response recommendations at different thresholds, assumed 1 hour per deployed response and an annual clinician workload = 1760 hours. NEWS2 were excluded from analysis in the presence of a DNACPR decision or when the patient was on ICU.

For peer review only

FIGURE LEGENDS

Figure 1 Modified consort diagram and overview of study design

Legend

a. Of 100,762 consecutive in-patient spells, 78,431 (77.8%) recorded at least one NEWS2 score whilst admitted outside of ICU. Of the remaining 21,931, 0.8% were admitted and discharged from ICU and so were never eligible for inclusion in the analysis. Of the others in which no NEWS2 score was recorded 96.8% had a length of stay (LOS) < 6 hours, 99.3% <12 hours (and 0.3% ≥24 hours). There were 29 deaths or admission to ICU associated with spells in which no NEWS2 was recorded.

b. Post-admission days were defined as consecutive 24-hour periods beginning at the time of admission T. The Index NEWS2 score was the first recorded in each of these periods at time t (●). A distinct overlapping 'patient-day' variable was then defined for each NEWS2 score between t and t + 24 hours during which, occurrence of a composite outcome event (◆) was recorded and linked to the peceeding Index NEWS2. Since the exact time *t* , that the Index-NEWS2 was recorded varies on each day, a small number of outcome events may be associated with no Index NEWS2 score (3.5%) as illustrated during Day 3, or with two Index NEWS2 scores (9.6%) as illustrated during Day 4.

Figure 2 Alert Rate and Number Needed to evaluate for NEWS2

Legend

a. Alert Rate for Index, All and Highest NEWS2 score vs sensitivity for Index NEWS2 score

The Alert Rate is the number of NEWS2 scores recorded at any given threshold, per 100 patients per post-admission day.

NEWS2 scores from Day 1 to 56 post-admission other than for Day 1 of elective admission were evaluated. The larger marker denotes the key action threshold NEWS2 ≥5, to the right of which is the marker for NEWS2 ≥5 or single parameter = 3 (and then NEWS2 ≥4, ≥3, ≥2, ≥1), and to left of which is NEWS2 ≥6 (and then NEWS2 ≥7, ≥8, ≥9, ≥10).

b. Number Needed to Evaluate vs sensitivity for Index NEWS2 score

The Number Needed to Evaluate is the number of patients to whom an Index NEWS2 score based clinical response must be deployed at threshold, to include one who then sustains a linked composite outcome event. The composite outcome event was defined as the first of unplanned admission to ICU (type 1 and 2 of the NHS critical care minimum dataset) or death of the patient within 24 hours of a NEWS2 score.

For peer review only - http://bmjopen.bmj.com/site/about/guidelines.xhtml

FUNDING

This work was funded by the HDR-UK better care and the PIONEER HDR-UK Hub in acute care, grant PIONEER2019.

DATA SHARING

The anonymised participant data and a data dictionary defining each field will be available to others through application to PIONEER, the HDR-UK Health data Hub via the corresponding author. The data will be available upon request and following approval by patients and public members (the PIONEER Data Trust Committee) and agreement of a process to ensure ethical data governance and through a data access licensing agreement. Please contact the corresponding author for details.

CONTRIBUTING STATEMENT

Pankhurst, Gkoutos and Ball designed the study. Gyves, Evison, Gallier curated the health data and conducted the analysis. Sapey and Ball wrote the paper. Pankhurst, Gallier, Gkoutos contributed to manuscript revision. All authors approved the final version.

COPYRIGHT AND LICENSE FOR PUBLICATION

The Corresponding Author has the right to grant on behalf of all authors and does grant on behalf of all authors, a worldwide licence to the Publishers and its licensees in perpetuity, in all forms, formats and media (whether known now or created in the future), to i) publish, reproduce, distribute, display and store the Contribution, ii) translate the Contribution into other languages, create adaptations, reprints, include within collections and create summaries, extracts and/or, abstracts of the Contribution, iii) create any other derivative work(s) based on the Contribution, iv) to exploit all subsidiary rights in the Contribution, v) the inclusion of electronic links from the Contribution to third party material where-ever it may be located; and, vi) licence any third party to do any or all of the above.

CONFLICTS OF INTEREST

For peer review only - http://bmjopen.bmj.com/site/about/guidelines.xhtml

TP, HG, FE, SG report no conflicts of interest. SB and GG report grant funding from HDR-UK during the conduct of the study. GG further acknowledges the support of the NIHR Birmingham ECMC, the NIHR Birmingham SRMRC and the Nanocommons H2020-EU (731032). ES reports grants from HDR-UK, during the conduct of the study; grants from Medical Research Council, grants from NIHR, grants from Wellcome Trust, grants from British Lung Foundation, grants from Alpha 1 Foundation, outside the submitted work.

For peer review only

REFERENCES

1. Royal College of Physicians. National Early Warning Score (NEWS) 2: Standardising the assessment of acute-illness severity in the NHS. Updated report of a working party. London: RCP, 2017.
2. NHS England, Royal College of Physicians, NHS Improvement. Patient safety alert: Resources to support the safe adoption of the revised National Early Warning Score (NEWS2). NHS, 2018.
3. Smith GB, Prytherch DR, Meredith P, et al. The ability of the National Early Warning Score (NEWS) to discriminate patients at risk of early cardiac arrest, unanticipated intensive care unit admission, and death. *Resuscitation*. 2013;84(4):465-70.
4. Alam N, Vegting IL, Houben E, et al. Exploring the performance of the National Early Warning Score (NEWS) in a European emergency department. *Resuscitation*. 2015;90:111-5.
5. Abbott TE, Vaid N, Ip D, et al. A single-centre observational cohort study of admission National Early Warning Score (NEWS). *Resuscitation*. 2015;92:89-93.
6. Kovacs C, Jarvis SW, Prytherch DR, et al. Comparison of the National Early Warning Score in non-elective medical and surgical patients. *Br J Surg*. 2016;103(10):1385-93.
7. Mellhammar L, Linder A, Tverring J, et al. Scores for sepsis detection and risk stratification - construction of a novel score using a statistical approach and validation of RETTS. *PLoS One*. 2020;15(2):e0229210.
8. Wuytack F, Meskell P, Conway A, et al. The effectiveness of physiologically based early warning or track and trigger systems after triage in adult patients presenting to emergency departments: a systematic review. *BMC Emerg Med*. 2017;17(1):38.
9. 2019 exceptional surveillance of sepsis: recognition, diagnosis and early management (NICE guideline NG51) and acutely ill adults in hospital: recognising and responding to deterioration (NICE guideline CG50). London: National Institute for Health and Care Excellence (UK). Copyright © NICE 2019.
10. Alam N, Hobbelink EL, van Tienhoven AJ, et al. The impact of the use of the Early Warning Score (EWS) on patient outcomes: a systematic review. *Resuscitation*. 2014;85(5):587-94.
11. Schmidt PE, Meredith P, Prytherch DR, et al. Impact of introducing an electronic physiological surveillance system on hospital mortality. *BMJ Quality & Safety*. 2015;24(1):10-20.
12. van Schalkwyk JM. Confounding explains 'deaths avoided'. *BMJ Quality & Safety*. 2015;24(2):175-.
13. Gao H, Harrison DA, Parry GJ, et al. The impact of the introduction of critical care outreach services in England: a multicentre interrupted time-series analysis. *Crit Care*. 2007;11(5):R113.
14. McNeill G, Bryden D. Do either early warning systems or emergency response teams improve hospital patient survival? A systematic review. *Resuscitation*. 2013;84(12):1652-67.
15. Gerry S, Bonnici T, Birks J, et al. Early warning scores for detecting deterioration in adult hospital patients: systematic review and critical appraisal of methodology. *BMJ*. 2020;369:m1501.
16. Critical care outreach teams. Emergency and acute medical care in over 16s: service delivery and organisation: NICE; 2018.
17. National Early Warning Score systems that alert to deteriorating adult patients in hospital (MIB 205). Medtech innovation briefing.: NICE; 2020.

For peer review only - http://bmjopen.bmj.com/site/about/guidelines.xhtml

18. Credland N, Dyson J, Johnson MJ. What are the patterns of compliance with Early Warning Track and Trigger Tools: A narrative review. *Appl Nurs Res*. 2018;44:39-47.
19. Hope J, Griffiths P, Schmidt PE, et al. Impact of using data from electronic protocols in nursing performance management: A qualitative interview study. *J Nurs Manag*. 2019;27(8):1682-90.
20. Paterson R, MacLeod DC, Thetford D, et al. Prediction of in-hospital mortality and length of stay using an early warning scoring system: clinical audit. *Clin Med (Lond)*. 2006;6(3):281-4.
21. Jarvis SW, Kovacs C, Briggs J, et al. Are observation selection methods important when comparing early warning score performance? *Resuscitation*. 2015;90:1-6.
22. Romero-Brufau S, Huddleston JM, Escobar GJ, et al. Why the C-statistic is not informative to evaluate early warning scores and what metrics to use. *Crit Care*. 2015;19:285.
23. Simmes F, Schoonhoven L, Mintjes J, et al. Financial consequences of the implementation of a rapid response system on a surgical ward. *J Eval Clin Pract*. 2014;20(4):342-7.
24. Pimentel MAF, Redfern OC, Gerry S, et al. A comparison of the ability of the National Early Warning Score and the National Early Warning Score 2 to identify patients at risk of in-hospital mortality: A multi-centre database study. *Resuscitation*. 2019;134:147-56.
25. Shamout F, Zhu T, Clifton L, et al. Early warning score adjusted for age to predict the composite outcome of mortality, cardiac arrest or unplanned intensive care unit admission using observational vital-sign data: a multicentre development and validation. *BMJ Open*. 2019;9(11):e033301.
26. Smith GB, Prytherch DR, Jarvis S, et al. A Comparison of the Ability of the Physiologic Components of Medical Emergency Team Criteria and the U.K. National Early Warning Score to Discriminate Patients at Risk of a Range of Adverse Clinical Outcomes. *Crit Care Med*. 2016;44(12):2171-81.
27. NHS Engalnd Seven Day Services Standards. 2017.
28. Kipnis P, Turk BJ, Wulf DA, et al. Development and validation of an electronic medical record-based alert score for detection of inpatient deterioration outside the ICU. *J Biomed Inform*. 2016;64:10-9.
29. Coleman JJ, van der Sijs H, Haefeli WE, et al. On the alert: future priorities for alerts in clinical decision support for computerized physician order entry identified from a European workshop. *BMC Med Inform Decis Mak*. 2013;13:111.
30. Redhead J. Personal communication. Time protocols for muting sepsis alerts when a clinical assessment indicates that a patient does not have sepsis but continues to have a high NEWS score. Results of a survey undertaken on behalf of the Shelford Group Medical Directors. 2019.
31. Escobar GJ, LaGuardia JC, Turk BJ, et al. Early detection of impending physiologic deterioration among patients who are not in intensive care: development of predictive models using data from an automated electronic medical record. *J Hosp Med*. 2012;7(5):388-95.
32. Murphy A, Cronin J, Whelan R, et al. Economics of Early Warning Scores for identifying clinical deterioration-a systematic review. *Ir J Med Sci*. 2018;187(1):193-205.
33. Hogan H, Hutchings A, Wulff J, et al. Interventions to reduce mortality from in-hospital cardiac arrest: a mixed-methods study. 2019;7:2.
34. McGinley A, Pearse RM. A national early warning score for acutely ill patients. *BMJ : British Medical Journal*. 2012;345:e5310.

For peer review only - http://bmjopen.bmj.com/site/about/guidelines.xhtml

35. Sepsis guidance implementation advice for adults. NHS England. London; 2017.
36. Corfield AR, Lees F, Zealley I, et al. Utility of a single early warning score in patients with sepsis in the emergency department. *Emerg Med J*. 2014;31(6):482-7.
37. Bonnici T, Gerry S, Wong D, et al. Evaluation of the effects of implementing an electronic early warning score system: protocol for a stepped wedge study. *BMC Medical Informatics and Decision Making*. 2016;16(1):19.
38. Escobar GJ, Liu VX, Schuler A, et al. Automated Identification of Adults at Risk for In-Hospital Clinical Deterioration. *N Engl J Med*. 2020;383(20):1951-60.

For peer review only

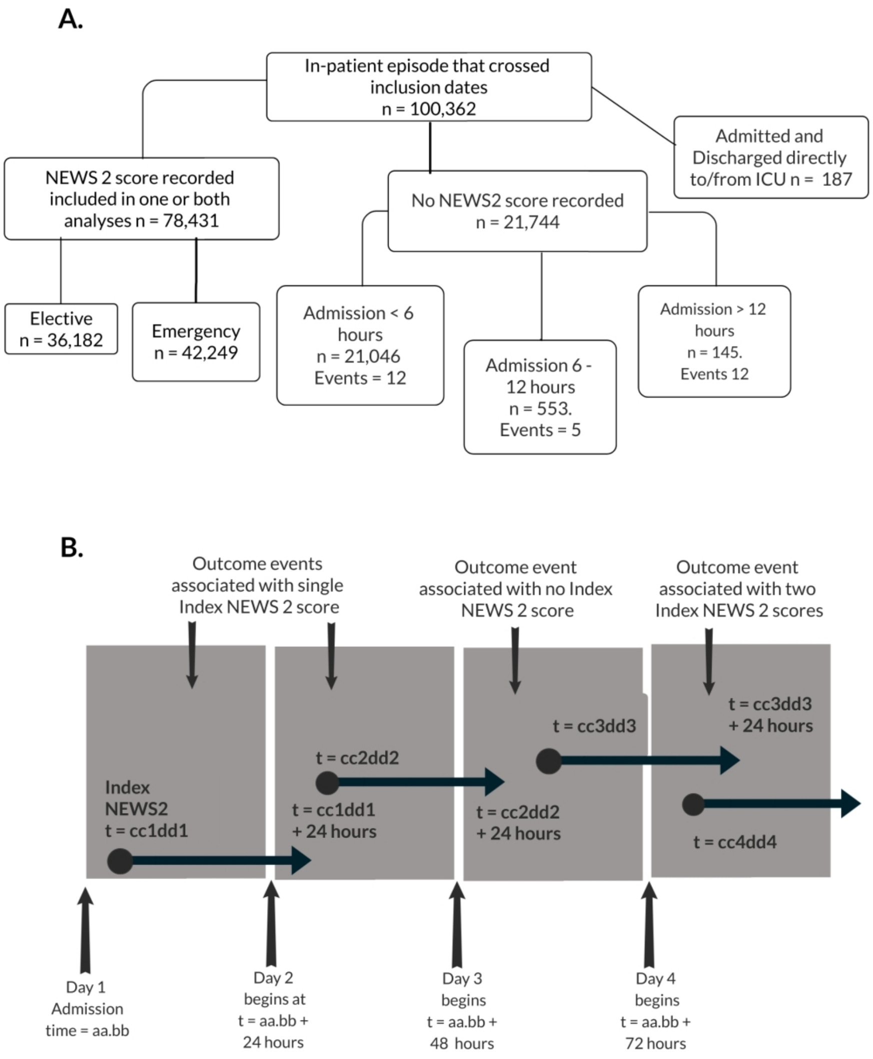

Figure 1 Modified consort diagram and overview of study design

Legend

a. Of 100,762 consecutive in-patient spells, 78,431 (77.8%) recorded at least one NEWS2 score whilst admitted outside of ICU. Of the remaining 21,931, 0.8% were admitted and discharged from ICU and so were never eligible for inclusion in the analysis. Of the others in which no NEWS2 score was recorded 96.8% had a length of stay (LOS) < 6 hours, 99.3% <12 hours (and 0.3% ≥24 hours). There were 29 deaths or admission to ICU associated with spells in which no NEWS2 was recorded.

b. Post-admission days were defined as consecutive 24-hour periods beginning at the time of admission T. The Index NEWS2 score was the first recorded in each of these periods at time t (black circle). A distinct overlapping 'patient-day' variable was then defined for each NEWS2 score between t and t + 24 hours during which, occurrence of a composite outcome event (black diamond) was recorded and linked to the preceeding Index NEWS2. Since the exact time t , that the Index-NEWS2 was recorded varies on each day, a small number of outcome events may be associated with no Index NEWS2 score (3.5%) as illustrated during Day 3, or with two Index NEWS2 scores (9.6%) as illustrated during Day 4.

831x1076mm (72 x 72 DPI)

Figure 2A

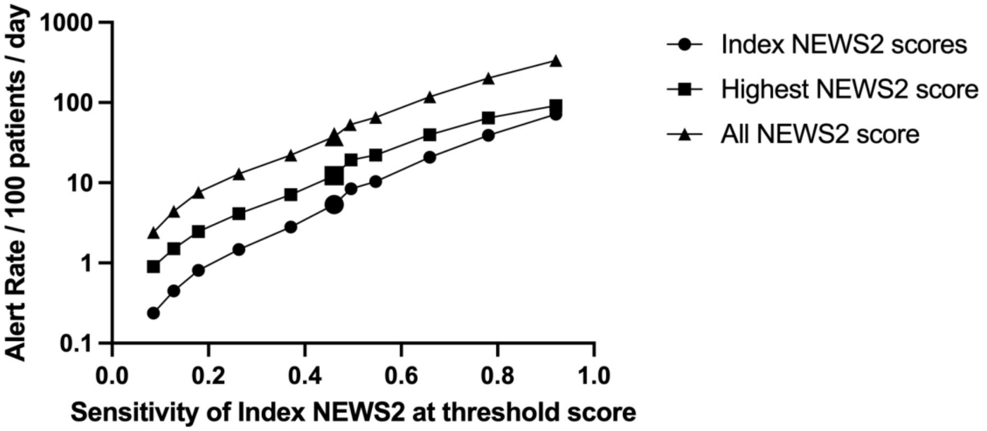

Figure 2B

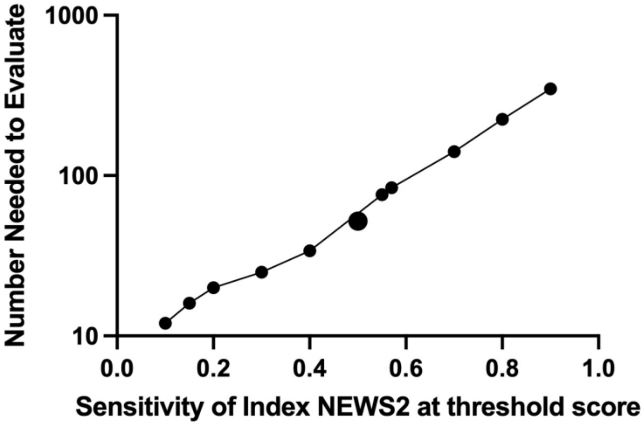

Figure 2 Alert Rate and Number Needed to evaluate for NEWS2

Legend

a. Alert Rate for Index, All and Highest NEWS2 score vs sensitivity for Index NEWS2 score

The Alert Rate is the number of NEWS2 scores recorded at any given threshold, per 100 patients per post-admission day.

NEWS2 scores from Day 1 to 56 post-admission other than for Day 1 of elective admission were evaluated. The larger marker denotes the key action threshold NEWS2 = or > 5, to the right of which is the marker for NEWS2 =or >5 or single parameter = 3 (and then NEWS2 = or > 4, = or > 3, = or >2, = or >1), and to left of which is NEWS2 = or > 6 (and then NEWS2 = or >7, = or >8, = or >9, = or >10).

b. Number Needed to Evaluate vs sensitivity for Index NEWS2 score

The Number Needed to Evaluate is the number of patients to whom an Index NEWS2 score based clinical response must be deployed at threshold, to include one who then sustains a linked composite outcome event. The composite outcome event was defined as the first of unplanned admission to ICU (type 1 and 2 of the NHS critical care minimum dataset) or death of the patient within 24 hours of a NEWS2 score.

158x175mm (300 x 300 DPI)

Online Supplement

Are current NEWS2 clinical response thresholds optimised for a general in-patient population?

Dr Tanya Pankhurst, Dr Elizabeth Sapey, Ms Helen Gyves, Ms Felicity Evison, Ms Suzy Gallier, Professor Georgios Gkoutos, Professor Simon Ball

For peer review only

Online Supplementary Figure S1: Post admission days with no Index NEWS2 recorded

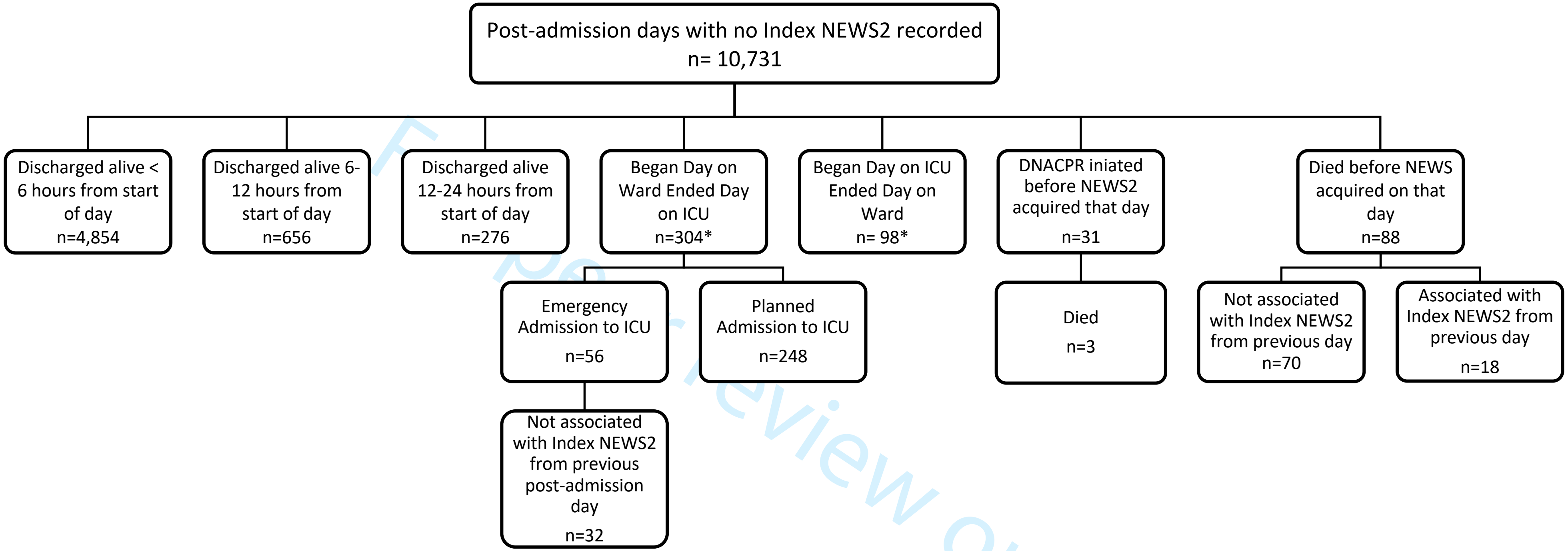

Legend. Of the 10,731 in which no Index-NEWS2 was recorded, 5,786 (53.9%) were discharged alive later that day. 433 (4.0%) were ineligible for part of the day because they were on ICU or had a DNACPR initiated. A further 88 (0.8%) died before a NEWS2 was recorded on that day. 4,424 post-admission days were therefore not associated with a NEWS2 record for unidentified reasons. There were 144 events (88 deaths and 56 emergency admissions to ICU) associated with post-admission days in which no Index-NEWS2 was recorded and another 3 in which a DNACPR was initiated prior to NEWS2 acquisition and subsequent death. Of the 144 events, 102 were not associated with an Index NEWS2 from the previous day, 20 because they occurred on the first day. The other 82 were associated with NEWS2 acquired later in the previous day but occurred > 24 hours after that day's Index-NEWS2.

For peer review only

Online Supplementary Figure S2

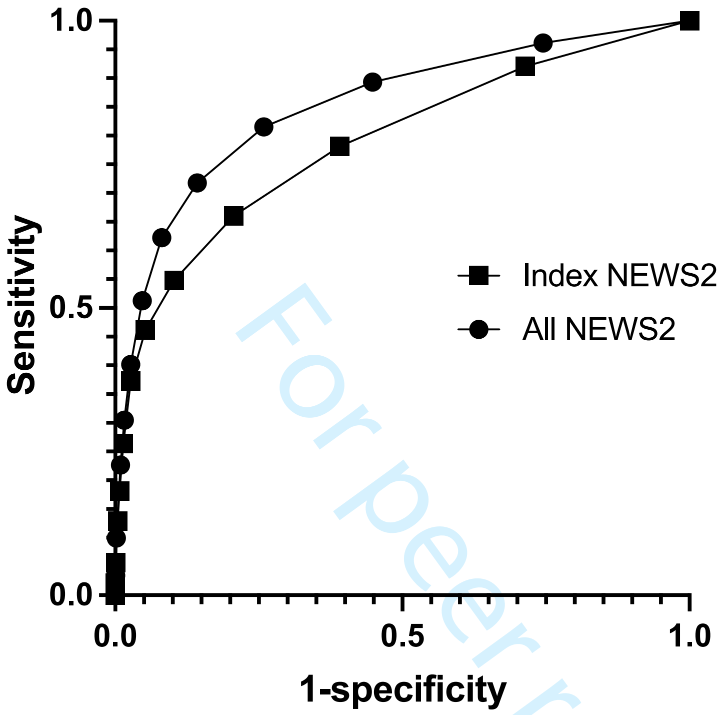

Legend

Receiver operating characteristic (ROC) for Index NEWS2 recorded in Day 1 to 56 excluding first post elective admission day (c statistic = 0.78, 95% CI:0.76-0.81) and All NEWS2 recorded in Days 1 to 56 excluding first post elective admission day (c statistic = 0.85, 95% CI: 0.85-0.86) $p<0.001$. A bootstrap analysis was performed with 10,000 repetitions of 10,000 patient-days, the median and 2.5th and 97.5th percentiles were then used to generate the ROC curve and corresponding confidence intervals. The outcome event was defined as the first of unplanned admission to ICU (type 1 and 2 of the NHS critical care minimum dataset) or death of the patient within 24 hours of a NEWS2 score.

For peer review only

Online Supplementary Figure S3a

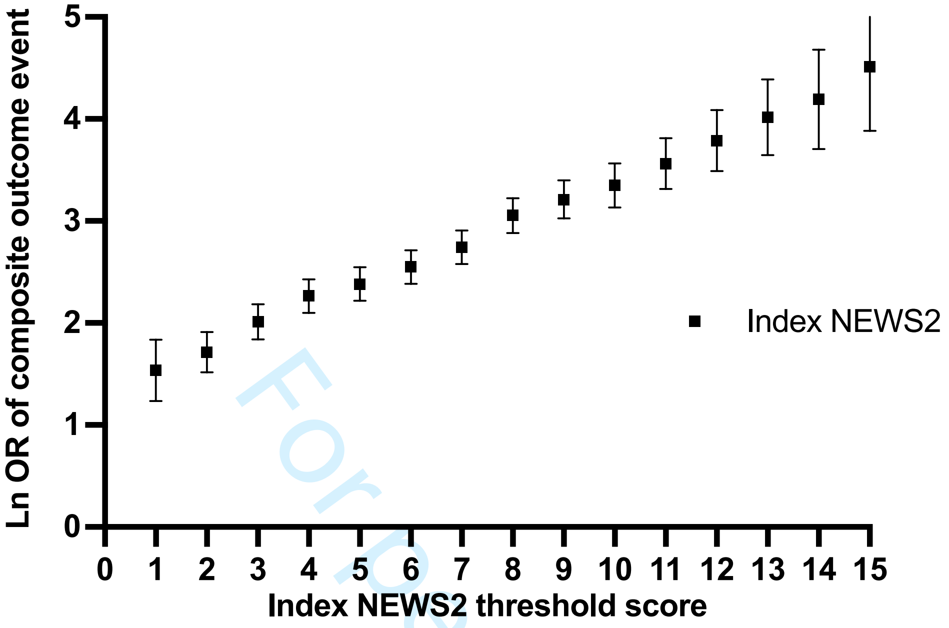

Legend

The Ln of the Odds Ratio (Ln OR) ± 95% confidence intervals for occurrence of the composite outcome event when the Index NEWS2 ≥ threshold score *vs.* < threshold score. The composite outcome event was the first of unplanned admission to ICU (type 1 and 2 of the NHS critical care minimum dataset) or death of the patient within 24 hours of a NEWS2 score. This analysis included NEWS2 scores from Day 1 to 56 post-admission other than for Day 1 of elective admission.

Although NEWS2 is not constituted to report absolute risk, it exhibits features valuable in representing risk. Integer changes in threshold were associated with approximately equivalent proportionate changes in the risk of an outcome event, across a wide range of scores and associated risk.

(These relationships resemble Fig 2b, because the relationship between sensitivity and Index NEWS2 is approximately negative linear and because at low event rates the odds of an event at threshold ≈ PPV = 1/NNE; the Ln (odds of an event) ≈ -Ln (NNE)).

For peer review only

Online Supplementary Figure S3b

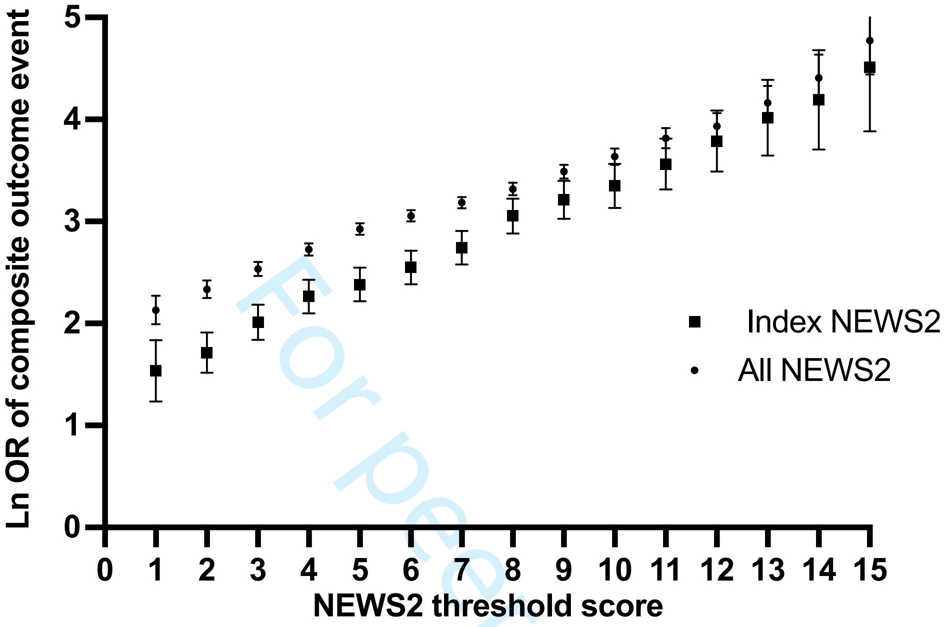

Legend

The Ln of the Odds Ratio (Ln OR) ± 95% confidence intervals for occurrence of the composite outcome event when the Index NEWS2 or All NEWS2 ≥ threshold score *vs.* < threshold score. This representation of the data reflects the differences in performance between Index-and All-NEWS2 shown in Fig S1.

For peer review only

Online Supplementary Tables
Online Supplementary Table S1 NEWS2 and SEWS scoring

National Early Warning Score (NEWS) 2 Standardising the assessment of acute-illness severity in the NHS– Royal College Physicians, London

Chart1: The NEWS2 scoring system

**Physiological parameter**				**Score**			
	3	2	1	0	1	2	3
Respiration rate (per minute)	≤8		9–11	12–20		21–24	≥25
SpO_2 Scale 1 (%)	≤91	92–93	94–95	≥96			
SpO_2 Scale 2 (%)	≤83	84–85	86–87	88–92 ≥93 on air	93–94 on oxygen	95–96 on oxygen	≥97 on oxygen
Air or oxygen?		Oxygen		Air			
Systolic blood pressure (mmHg)	≤90	91–100	101–110	111–219			≥220
Pulse (per minute)	≤40		41–50	51–90	91–110	111–130	≥131
Consciousness				Alert			CVPU
Temperature (°C)	≤35.0		35.1-36.0	36.1-38.0	38.1-39.0	≥39.1	

Chart 2: NEWS2 thresholds and triggers

**NEW score Clinical risk Response**		
Aggregate score 0–4	Low	Ward-based response
Red score<br>Score of 3 in any individual parameter	Low–medium	Urgent ward-based response*
Aggregate score 5–6	Medium	Key threshold for urgent response*
Aggregate score 7 or more	High	Urgent or emergency response**

* Response by a clinician or team with competence in the assessment and treatment of acutely ill patients and in recognising when the escalation of care to a critical care team is appropriate.
**The response team must also include staff with critical care skills, including airway management.
National Early Warning Score (NEWS) 2 Standardising the assessment of acute-illness severity in the NHS– Royal College Physicians, London,
© Royal College of Physicians 2017

Standardised early warning scoring system (SEWS) parameters and scoring system.

**Physiological parameter**				**Score**			
	3	2	1	0	1	2	3
Respiration rate (per minute)	≤8			9–20	21-30	31-35	≥36
SaO_2	<85	85-89	90-92	≥93			
Temperature (°C)	≤33.9	34.0-34.9	35.0-35.9	36.0-37.9	38.0-38.9	≥39.0	
Blood pressure (mmHg)	≤69	70-79	80-99	100-199		≥200	
Pulse (per minute)	≤29	30-39	40-49	50-99	100-109	110-129	≥130
AVPU				Alert	Verbal	Pain	None

For peer review only

Supplementary Table 2 – Index NEWS2 and SEWS (a) and linked outcomes events (b) from Day 1 to 56 post-admission excluding Day 1 of elective admission

Table S2a Index NEWS2 and SEWS

		NEWS2																				
		0	1	2	3	4	3 single	4 single	5	6	7	8	9	10	11	12	13	14	15	16	17	Total SEWS
SEWS	0	72852	54298	21005	6960	2310	123	110	891	232	40	5	1	1	0	0	0	0	0	0	0	158828
	1	674	28195	22333	10054	3685	2628	1565	2212	867	292	57	19	3	1	1	0	0	0	0	0	72586
	2	283	835	3995	4771	2162	1029	1512	1910	952	427	174	60	16	3	1	0	0	0	0	0	18130
	3	2	170	291	800	925	445	478	1068	793	481	246	102	35	17	4	0	0	0	0	0	5857
	4	0	5	27	43	156	9	123	394	431	301	211	143	68	15	7	2	2	0	0	0	1937
	5	0	0	0	4	10	3	14	62	129	143	157	118	85	39	16	4	6	0	1	0	791
	6	0	0	0	0	0	0	0	5	21	40	66	66	59	46	23	10	2	0	0	0	338
	7	0	0	0	0	0	0	0	0	1	5	15	27	24	26	12	8	2	2	1	0	123
	8	0	0	0	0	0	0	0	0	2	0	6	7	7	10	10	5	4	3	1	1	56
	9	0	0	0	0	0	0	0	0	0	0	0	2	2	2	4	4	2	2	0	0	18
	10	0	0	0	0	0	0	0	0	0	0	0	0	0	2	1	2	1	0	1	2	9
	11	0	0	0	0	0	0	0	0	0	0	0	0	0	1	0	1	0	0	1	0	3
	12	0	0	0	0	0	0	0	0	0	0	0	0	0	0	0	0	0	1	0	0	1
	13	0	0	0	0	0	0	0	0	0	0	0	0	0	0	0	0	0	0	0	1	1
	Total	73811	83503	47651	22632	9248	4237	3802	6542	3428	1729	937	545	300	162	79	36	19	8	5	4	

Of the 8039 that NEWS2 = 3 in any single parameter 149 (1.8%) triggered the lowest SEWS threshold ≥4
Of the 6542 that NEWS2 = 5, 461 triggered (7.0%) the lowest SEWS threshold ≥4
Of the 3428 that NEWS2 = 6, 584 triggered (16.0%) the lowest SEWS threshold ≥4

For peer review only

Table S2b outcome events linked to Index NEWS2 and SEWS

		NEWS2																				
		0	1	2	3	4	3 single	4 single	5	6	7	8	9	10	11	12	13	14	15	16	17	Total SEWS
	0	45	52	28	21	9	0	0	3	2	1	0	0	0	0	0	0	0	0	0	0	161
	1	0	25	30	19	19	5	3	12	18	5	0	1	0	0	0	0	0	0	0	0	137
	2	1	3	11	11	7	2	5	20	16	6	5	3	1	0	1	0	0	0	0	0	92
	3	0	1	1	4	5	3	1	14	16	20	8	7	0	0	1	0	0	0	0	0	81
	4	0	0	0	0	0	0	1	3	8	7	5	4	1	1	0	0	0	0	0	0	30
SEWS	5	0	0	0	0	0	0	0	0	2	8	9	6	8	4	3	0	2	0	0	0	42
	6	0	0	0	0	0	0	0	0	1	1	3	1	6	4	2	1	0	0	0	0	19
	7	0	0	0	0	0	0	0	0	0	0	0	0	2	5	0	2	0	0	0	0	9
	8	0	0	0	0	0	0	0	0	0	0	0	2	0	0	0	1	2	1	1	0	7
	9	0	0	0	0	0	0	0	0	0	0	0	0	0	0	0	1	0	0	0	0	1
	10	0	0	0	0	0	0	0	0	0	0	0	0	0	0	0	1	0	0	0	0	1
	11	0	0	0	0	0	0	0	0	0	0	0	0	0	0	0	0	0	0	0	0	0
	12	0	0	0	0	0	0	0	0	0	0	0	0	0	0	0	0	0	0	0	0	0
	13	0	0	0	0	0	0	0	0	0	0	0	0	0	0	0	0	0	0	0	0	0
	Total	46	81	70	55	40	10	10	52	63	48	30	24	18	14	7	6	4	1	1	0	

Of the 20 that NEWS2 = 3 in any single parameter, 1 (5.0%) triggered the lowest SEWS threshold ≥4
Of the 52 that NEWS2 = 5, 3 (5.8%) the lowest SEWS threshold ≥4
Of the 63 that NEWS2 = 6, 11 triggered (17.5%) the lowest SEWS threshold ≥4

For peer review only

Supplementary Table 3 – All NEWS2 and SEWS (a) and linked outcomes events (b) from Day 1 to 56 post-admission excluding Day 1 of elective admission

Table S3a All NEWS2 and SEWS

		NEWS2																					
		0	1	2	3	4	3 single	4 single	5	6	7	8	9	10	11	12	13	14	15	16	17	18	Total SEWS
	0	291135	225784	99131	37585	12886	631	500	4720	1444	269	46	11	1	0	0	0	0	0	0	0	0	674143
	1	2748	114393	99833	49029	20759	12435	7958	13629	6182	2206	696	143	34	5	1	0	0	0	0	0	0	330051
	2	1159	3772	18598	24228	12226	4894	7661	11932	7365	3669	1681	666	175	37	11	1	0	0	0	0	0	98075
	3	10	583	1364	3940	5404	2053	2820	6782	5438	3983	2413	1231	462	188	54	12	2	0	0	0	0	36739
	4	0	15	113	279	778	55	653	2462	2560	2349	1883	1428	768	302	122	33	9	3	0	0	0	13812
SEWS	5	0	0	2	19	53	11	44	369	809	1021	1044	1024	763	442	184	76	28	9	1	0	0	5899
	6	0	0	0	0	2	0	3	15	105	227	365	503	485	368	213	112	48	12	3	0	0	2461
	7	0	0	0	0	0	0	0	1	13	27	83	166	158	197	151	89	59	17	8	0	2	971
	8	0	0	0	0	0	0	0	0	3	4	19	30	62	98	64	64	40	22	9	2	1	418
	9	0	0	0	0	0	0	0	0	0	0	3	5	13	19	40	35	22	20	11	3	0	171
	10	0	0	0	0	0	0	0	0	0	0	0	2	2	4	11	10	12	6	9	4	1	61
	11	0	0	0	0	0	0	0	0	0	0	0	0	0	1	1	3	2	2	4	0	0	13
	12	0	0	0	0	0	0	0	0	0	0	0	0	0	0	0	1	3	2	1	1	0	8
	13	0	0	0	0	0	0	0	0	0	0	0	0	0	0	0	0	0	1	0	1	0	2
	Total	295052	344547	219041	115080	52108	20079	19639	39910	23919	13755	8233	5209	2923	1661	852	436	225	94	46	11	3	1,162,824

Of the 39178 that NEWS2 = 3 in any single parameter, (1.9%) 766 triggered the lowest SEWS threshold ≥4
Of the 39910 that NEWS2 = 5, 2847 triggered (7.1%) the lowest SEWS threshold ≥4
Of the 23919 that NEWS2 = 6, 3490 triggered (14.5%) the lowest SEWS threshold ≥4

For peer review only

Table S3b outcome events linked to All NEWS2 and SEWS

		NEWS2																					
		0	1	2	3	4	3 single	4 single	5	6	7	8	9	10	11	12	13	14	15	16	17	18	Total SEWS
SEWS	0	200	208	194	150	73	0	1	37	16	5	0	0	0	0	0	0	0	0	0	0	0	884
	1	3	140	162	163	152	39	29	168	108	56	16	4	0	0	0	0	0	0	0	0	0	1040
	2	3	7	55	94	81	30	63	194	188	117	71	37	14	1	2	0	0	0	0	0	0	957
	3	0	2	3	23	55	15	31	135	159	189	130	80	32	16	7	1	0	0	0	0	0	878
	4	0	0	0	2	5	0	12	40	89	96	108	118	62	47	14	1	1	0	0	0	0	595
	5	0	0	0	0	0	0	0	6	24	40	60	81	88	63	23	13	7	2	0	0	0	407
	6	0	0	0	0	0	0	0	0	2	7	24	50	54	54	23	14	5	3	0	0	0	236
	7	0	0	0	0	0	0	0	0	0	1	3	17	24	31	30	17	11	6	1	0	2	143
	8	0	0	0	0	0	0	0	0	0	2	1	5	3	11	14	19	14	11	4	1	0	85
	9	0	0	0	0	0	0	0	0	0	0	0	0	2	1	4	10	5	6	6	0	0	34
	10	0	0	0	0	0	0	0	0	0	0	0	1	0	0	4	2	3	4	3	1	1	19
	11	0	0	0	0	0	0	0	0	0	0	0	0	0	0	0	0	1	0	3	0	0	4
	12	0	0	0	0	0	0	0	0	0	0	0	0	0	0	0	1	1	0	0	0	0	2
	13	0	0	0	0	0	0	0	0	0	0	0	0	0	0	0	0	0	0	0	0	0	0
	Total	206	357	414	432	366	84	136	580	586	513	413	393	279	224	121	78	48	32	17	2	2	5284

Of the 220 that NEWS2 = 3 in any single parameter 12 (5.4%) triggered the lowest SEWS threshold ≥4
Of the 580 that NEWS2 = 5, 46 triggered (7.9%) the lowest SEWS threshold ≥4
Of the 586 that NEWS2 = 6, 115 triggered (19.6%) the lowest SEWS threshold ≥4

5284 outcome events are recorded because every NEWS2 is treated independently and linked to an outcome event over the subsequent 24 hours.

For peer review only

Supplementary Table 4 bed occupancy by the in-patient population at midday

Group	Number of Beds occupied at 12.00[a]
**Patients eligible for NEWS2 analysis**	
emergency admission in hospital < 24 hours at 12.00	98 ± 15
emergency admission in hospital ≥24 hours at 12.00	632 ± 31
elective admission in hospital <24 hours at 12.00	150 (IQR 53-162)
elective admission in hospital ≥24 hours at 12.00	198 ± 21
**Patients not eligible for NEWS2 analysis**	
ICU	72 (IQR: 67-76)
DNACPR	170 ± 15

Supplementary Table 4 legend

Number of beds occupied at midday for the 273 days between 01/11/2018 and 31/7/2019.

[a] Mean ± standard deviation for normally distributed groups. Median and interquartile range if not normally distributed. (Elective admission in hospital <24 hours included day case admissions which vary significantly with the day of the week). In those eligible for NEWS2 analysis, weekday bed occupancy = 1096 (median; IQR: 1079-1113) and weekend bed occupancy = 949 (median; IQR: 937-976). Across all days, the mean daily bed occupancy at midday in those eligible for NEWS2 analysis = 1046.

For peer review only - http://bmjopen.bmj.com/site/about/guidelines.xhtml

STROBE Statement—Checklist of items that should be included in reports of ***cohort studies***

	Item No	Recommendation	Response
**Title and abstract**	1	(*a*) Indicate the study's design with a commonly used term in the title or the abstract	Yes. Pg 3
		(*b*) Provide in the abstract an informative and balanced summary of what was done and what was found	Yes. Pg 3
**Introduction**			
Background/rationale	2	Explain the scientific background and rationale for the investigation being reported	Yes. Pg 5
Objectives	3	State specific objectives, including any prespecified hypotheses	Yes. Pg 5
**Methods**			
Study design	4	Present key elements of study design early in the paper	Yes. Pg 6
Setting	5	Describe the setting, locations, and relevant dates, including periods of recruitment, exposure, follow-up, and data collection	Yes. Pg 6
Participants	6	(*a*) Give the eligibility criteria, and the sources and methods of selection of participants. Describe methods of follow-up	Yes. Pg 6-7
		(*b*) For matched studies, give matching criteria and number of exposed and unexposed	N/A
Variables	7	Clearly define all outcomes, exposures, predictors, potential confounders, and effect modifiers. Give diagnostic criteria, if applicable	Yes Pg 6-7
Data sources/ measurement	8*	For each variable of interest, give sources of data and details of methods of assessment (measurement). Describe comparability of assessment methods if there is more than one group	Yes Pg 6-7
Bias	9	Describe any efforts to address potential sources of bias	Yes Pg 6-7
Study size	10	Explain how the study size was arrived at	Yes. Pg 6-7
Quantitative variables	11	Explain how quantitative variables were handled in the analyses. If applicable, describe which groupings were chosen and why	Yes. Pg 6-7
Statistical methods	12	(*a*) Describe all statistical methods, including those used to control for confounding	Yes Pg 8-9
		(*b*) Describe any methods used to examine subgroups and interactions	Yes Pg 8-9
		(*c*) Explain how missing data were addressed	Yes Pg 8-9
		(*d*) If applicable, explain how loss to follow-up was addressed	N/A
		(*e*) Describe any sensitivity analyses	Yes Pg 8-9
**Results**			
Participants	13*	(a) Report numbers of individuals at each stage of study—eg numbers potentially eligible, examined for eligibility, confirmed eligible, included in the study, completing follow-up, and analysed	Yes Pg 10
		(b) Give reasons for non-participation at each stage	Yes Pg 10
		(c) Consider use of a flow diagram	Yes Fig 1
Descriptive data	14*	(a) Give characteristics of study participants (eg demographic, clinical, social) and information on exposures and potential confounders	Yes Table 1
		(b) Indicate number of participants with missing data for each variable of interest	Yes Figure 1
		(c) Summarise follow-up time (eg, average and total amount)	Yes Pg 10
Outcome data	15*	Report numbers of outcome events or summary measures over time	Yes Pg 10
Main results	16	(*a*) Give unadjusted estimates and, if applicable, confounder-adjusted estimates and their precision (eg, 95% confidence interval). Make clear	Yes Pg 11 - 12

For peer review only - http://bmjopen.bmj.com/site/about/guidelines.xhtml

		which confounders were adjusted for and why they were included	
		(*b*) Report category boundaries when continuous variables were categorized	Yes Pg 11 - 12
		(*c*) If relevant, consider translating estimates of relative risk into absolute risk for a meaningful time period	Yes Pg 11 -1 2
Other analyses	17	Report other analyses done—eg analyses of subgroups and interactions, and sensitivity analyses	Yes Pg 11 - 12
**Discussion**			
Key results	18	Summarise key results with reference to study objectives	Yes. Pg 14
Limitations	19	Discuss limitations of the study, taking into account sources of potential bias or imprecision. Discuss both direction and magnitude of any potential bias	Yes Pg 16
Interpretation	20	Give a cautious overall interpretation of results considering objectives, limitations, multiplicity of analyses, results from similar studies, and other relevant evidence	Yes Pg 16
Generalisability	21	Discuss the generalisability (external validity) of the study results	Yes Pg 16
**Other information**			
Funding	22	Give the source of funding and the role of the funders for the present study and, if applicable, for the original study on which the present article is based	Yes Pg 17

*Give information separately for exposed and unexposed groups.

Note: An Explanation and Elaboration article discusses each checklist item and gives methodological background and published examples of transparent reporting. The STROBE checklist is best used in conjunction with this article (freely available on the Web sites of PLoS Medicine at http://www.plosmedicine.org/, Annals of Internal Medicine at http://www.annals.org/, and Epidemiology at http://www.epidem.com/). Information on the STROBE Initiative is available at http://www.strobe-statement.org.

For peer review only

BMJ Open

An evaluation of NEWS2 response thresholds in a retrospective observational study from a UK acute hospital.

Journal:	*BMJ Open*
Manuscript ID	bmjopen-2021-054027.R1
Article Type:	Original research
Date Submitted by the Author:	20-Nov-2021
Complete List of Authors:	Pankhurst, Tanya ; University Hospitals Birmingham NHS Foundation Trust,<br>Sapey, Elizabeth; University of Birmingham, Institute of Inflammation and Ageing<br>Gyves, Helen; University Hospitals Birmingham NHS Foundation Trust, Health Informatics<br>Evison, Felicity; University Hospitals Birmingham NHS Foundation Trust, Department of Health Informatics<br>Gallier, Suzy; University Hospitals Birmingham NHS Foundation Trust, Health Informatics; University of Birmingham, PIONEER Technical Director<br>Gkoutos, George; University of Birmingham,<br>Ball, Simon; University Hospitals Birmingham NHS Foundation Trust; Health Data Research UK, Better Care
**Primary Subject Heading**:	Health policy
Secondary Subject Heading:	Health informatics, Medical management
Keywords:	INTERNAL MEDICINE, Health policy < HEALTH SERVICES ADMINISTRATION & MANAGEMENT, Health informatics < BIOTECHNOLOGY & BIOINFORMATICS

SCHOLARONE™
Manuscripts

I, the Submitting Author has the right to grant and does grant on behalf of all authors of the Work (as defined in the below author licence), an exclusive licence and/or a non-exclusive licence for contributions from authors who are: i) UK Crown employees; ii) where BMJ has agreed a CC-BY licence shall apply, and/or iii) in accordance with the terms applicable for US Federal Government officers or employees acting as part of their official duties; on a worldwide, perpetual, irrevocable, royalty-free basis to BMJ Publishing Group Ltd ("BMJ") its licensees and where the relevant Journal is co-owned by BMJ to the co-owners of the Journal, to publish the Work in this journal and any other BMJ products and to exploit all rights, as set out in our licence.

The Submitting Author accepts and understands that any supply made under these terms is made by BMJ to the Submitting Author unless you are acting as an employee on behalf of your employer or a postgraduate student of an affiliated institution which is paying any applicable article publishing charge ("APC") for Open Access articles. Where the Submitting Author wishes to make the Work available on an Open Access basis (and intends to pay the relevant APC), the terms of reuse of such Open Access shall be governed by a Creative Commons licence – details of these licences and which Creative Commons licence will apply to this Work are set out in our licence referred to above.

Other than as permitted in any relevant BMJ Author's Self Archiving Policies, I confirm this Work has not been accepted for publication elsewhere, is not being considered for publication elsewhere and does not duplicate material already published. I confirm all authors consent to publication of this Work and authorise the granting of this licence.

For peer review only

An Evaluation of NEWS2 response thresholds in a retrospective observational study from a UK acute hospital

Dr Tanya Pankhurst[1], Dr Elizabeth Sapey[2], Ms Helen Gyves[3], Ms Felicity Evison[4], Ms Suzy Gallier[5], Professor Georgios Gkoutos[6], Professor Simon Ball[1, 7]

Affiliations

1. University Hospitals Birmingham NHS Foundation Trust, Edgbaston, Birmingham, UK Tanya.Pankhurst@uhb.nhs.uk

2. Corresponding author.

a. PIONEER, HDRUK Health Data Hub in Acute Care, Birmingham, UK. University of Birmingham, Edgbaston, Birmingham, UK, B15 2GW. Institute of Inflammation and Ageing, University of Birmingham, Birmingham, UK. ORCiD ID: 0000-0003-3454-5482. Email: e.sapey@bham.ac.uk Tel: 00 44 121 246 2000

b. Acute Medicine, University Hospitals Birmingham NHS Foundation Trust, Edgbaston, Birmingham, UK.

3. University Hospitals Birmingham NHS Foundation Trust, Edgbaston, Birmingham, UK. Helen.Gyves@UHB.nhs.uk

4. University Hospitals Birmingham NHS Foundation Trust, Edgbaston, Birmingham, UK. Felicity.Evison@UHB.nhs.uk

5. University Hospitals Birmingham NHS Foundation Trust, Edgbaston, Birmingham, UK. Suzy.Gallier@UHB.nhs.uk

6. Health Data Research UK (HDR-UK) Midland's Physical Site, Birmingham, UK. Institute of Cancer and Genomic Sciences, University of Birmingham, Birmingham, UK. G.Gkoutos@bham.ac.uk

7. Health Data Research UK (HDR-UK) Midland's Physical Site. University of Birmingham, Edgbaston, Birmingham, UK. Institute of Immunology and Immunotherapy, University of Birmingham, Birmingham, UK. simon.ball@uhb.nhs.uk

For peer review only

Funding

Health Data Research UK.

Key words

Abstract word count: 299

Main article word count: 4095 (excluding citations)

References: 43

Tables: 3

Figures: 2

There is an online supplement

peer review only

ABSTRACT

Objective - Use of National Early Warning Score 2 (NEWS2) has been mandated in adults admitted to acute hospitals in England. Urgent clinical review is recommended at NEWS2≥5. This policy is recognised as requiring ongoing evaluation. We assessed NEWS2 acquisition, alerting at key thresholds, and patient outcomes, to understand how response recommendations would affect clinical resource allocation.

Setting. Adult acute hospital in England.

Design. Retrospective observational cohort study.

Participants. 100,362 consecutive admissions between Nov 2018-Jul 2019.

Outcome. Death or admission to Intensive Care Unit (ICU) within 24 hours of a score.

Methods. NEWS2 were assembled as single scores from consecutive 24-hour timeframes, (the first NEWS2 termed "Index-NEWS2"), or as all scores from the admission (termed All-NEWS2). Scores were excluded when a patient was in intensive care, in the presence of a decision not to attempt cardiopulmonary resuscitation (DNACPR), or on Day 1 of elective admission.

Results. A mean of 4.5 NEWS2 were acquired per patient per day. The outcome rate following an Index-NEWS2 was 0.22/100 patient-days. The sensitivity of outcome prediction at Index-NEWS2 ≥5 = 0.46, and number needed to evaluate (NNE) = 52. At this threshold, a mean of 37.6 alerts /100 patient-days would be generated, occurring in 12.3% of patients on any single day. Threshold changes to increase sensitivity by 0.1, would result in a 2-fold increase in alert rate and 1.5-fold increase in NNE. Overall, NEWS2 classification performance was significantly worse on Index-scores than All-scores (c-statistic = 0.78 vs 0.85; p<0.001).

Conclusions. The combination of low event-rate, high alert-rate and low sensitivity, in patients for cardiopulmonary resuscitation, means that at current NEWS2 thresholds, resource demand would be sufficient to meaningfully compete with other pathways to clinical evaluation. In analyses that epitomise in-patient screening, NEWS2 performance suggests a need for re-evaluation of current response recommendations in this population.

For peer review only

Strengths and limitations of the study

- All admissions to an acute hospital within the study timeframe are included, providing the basis for a detailed understanding of the consequences of NEWS2 based policy.
- A precise definition of DNACPR decisions over the course of an admission informed our analysis, in order to maintain correspondence with decision making and treatment options available clinically.
- The evaluation of NEWS2 as a single score from discrete 24-hour timeframes, provides an assessment of classification performance which epitomises its role in screening patients between routine reviews, moderating the effects of including large numbers of scores acquired from those already identified to be at clinical risk.
- The analysis is limited by the fact that this is a single centre study, however the underlying data are consistent with other reports from acute hospitals in the NHS.
- The analysis excludes Day 1 of elective admission from outcome-based analysis, because deranged physiology was predicted to occur following planned intervention on that day.

For peer review only

INTRODUCTION

The use of early warning scores (EWS's) has been widely advocated, to integrate physiological parameters into a single actionable output. In 2017, the Royal College of Physicians (RCP) published a modified National Early Warning Score (NEWS), referred to as NEWS2 [1]. NEWS2 is a scoring system based upon six physiological parameters. It is associated with specific clinical response recommendations, including urgent clinical review at a key threshold NEWS2 score ≥5. This requires attendance by clinicians with competence in the assessment and treatment of acutely ill patients, and where necessary, escalation to a team with critical care competencies [1]. At NEWS2 score ≥7, this is uplifted to assessment by a team with those critical care competencies. In 2019, the National Health Service in England (NHSE) mandated the use of NEWS2 for all adults in acute hospitals and widened its application to include screening for sepsis [2]. This extends its use significantly beyond the evaluation of acute admissions, for which NEWS was initially developed and validated [3-8]. The National Institute for Health and Care Excellence (NICE) has identified the need for further evaluation of NEWS2, to ensure no adverse consequences from its roll out across the NHS [9]. This is in the context of limited evidence of survival benefit from an EWS triggered response [8, 10-16], or definition of the resource required to deliver a response [17]. Since electronic systems directly link a threshold EWS to a response recommendation [18], high rates of alerting may arise, unmoderated by human intervention [19, 20], creating an opportunity for clinical resource to be diverted in ways that could be counterproductive [9].

Given a requirement to significantly alter clinical practice [1, 2], we evaluated the performance of NEWS2, across the in-patient population of an acute hospital. This included a description of the rate of NEWS2 data acquisition, the rates at which key threshold scores were met, and their relationship to outcome. This was with a view to understanding how NEWS2 based recommendations could affect the distribution of resource when put into practice, taking into account factors which may modify the outcome, such as resuscitation status, or those which may amplify demand, such as recurrent alerting [9].

METHODS

Setting

The Queen Elizabeth Hospital Birmingham (QEHB) is a National Health Service (NHS), urban, adult, acute hospital in England with 1269 beds including 80 level 2/3 ICU beds, an Emergency Department that assesses >300 patients per day, and a mixed secondary and tertiary practice that includes all major adult specialities with the exception of obstetrics and gynaecology. The EHR at QEHB (PICS, Birmingham Systems) contains time-stamped, structured records that include demography, location, time of admission and discharge, physiological measurements supporting NEWS2 and Standard Early Warning Score (SEWS) (See Table S1 of the online supplement) and Do Not Attempt Cardiopulmonary Resuscitation (DNACPR) decisions subject to regular review underpinned by the clinical decision support system. NEWS2 observations were recorded by trained healthcare staff with devices used and maintained in accordance with the Medicines and Healthcare Products Regulatory Agency guidance [21].

This study was approved by the East Midlands–Derby Research Ethics Committee (reference: 20/EM/0158) and Confidentiality Advisory Group (CAG20/CAG/0084) as part of PIONEER, the HDR-UK Hub in Acute Care.

NEWS2 data collection was electronically mandated, whilst alerting continued to use established SEWS thresholds [22], (coincidentally facilitating an assessment of NEWS2 performance at the key action threshold ≥5, minimally disrupted by triggered clinical responses (See Table S2a and S2b and Table S3a and Table S3b of the online supplement)). Existing standards included a maximum interval between EWS acquisition = 12 hours, progressive alerting to ward-based staff and automated escalation to a 24/7 critical care outreach team at threshold SEWS.

Cohort and Definitions

All hospital spells (continuous stay in a hospital bed), between 00.00 on 01/11/2018 and 23.59 on 31/7/2019, were evaluated to discharge (99.6%), or to 56 days post admission (0.4%) if that was earlier. Initial Emergency Department assessments, prior to admission, were not included. An adapted consort diagram is shown in Figure 1a. Admissions were identified as emergency or elective from the mandatory provider spell admission method code. A composite outcome event

was defined as the first of unplanned admission to ICU (type 1 and 2 of the NHS critical care minimum dataset [23]) or death of the patient, within 24 hours of a NEWS2 score.

In the primary analysis, each spell was divided into consecutive 24-hour post-admission days starting at the time of admission. As shown in Figure 1b, for admission time T, consecutive 24-hour periods end at T + (n x 24 hours); where n is the nominal post-admission day. The first NEWS2 recorded in each post-admission day, at time t_n was termed the Index-NEWS2 score. An overlapping, patient-day variable was defined between t_n and t_n+24hours. The first outcome occurring in the patient-day was linked to the preceding Index-NEWS2. This design was used to ensure a single NEWS2 score was captured from discrete, consecutive, 24-hour time periods and that the outcome was assessed over 24 hours.

In addition to Index-NEWS2, analyses were undertaken using all the scores recorded in a given time period (termed All-NEWS2) [24] and using the highest score on each post-admission day (termed Highest-NEWS2). NEWS2 scores were not eligible for inclusion if at the time the score was acquired, the patient was in ICU or had a DNACPR in place. This was to achieve internal consistency when using admission to ICU as part of the composite outcome, since decisions not to resuscitate are highly concordant with ceiling of care not including ICU. On return from ICU or if a DNACPR was revoked, subsequent scores were included in the analysis. No score was calculated when patients underwent an operative procedure away from the ward.

Day 1 post elective-admission was excluded from outcome-based analysis because it was known that almost all were admitted for surgery later that day, after a NEWS2 was recorded. Any relationship with outcome might then be confounded by decisions not to proceed to intervention on that day, informed by NEWS2 or its component observations. All other patient-days were eligible for analysis.

Patient demographics are reported for the first admission in the study period. Additional context is provided by reporting bed occupancy at midday across the study period (See Table S4 of the online supplement).

Analysis of EWS performance

For peer review only - http://bmjopen.bmj.com/site/about/guidelines.xhtml

To evaluate the performance of different NEWS2 threshold scores, a patient with a NEWS2 ≥ threshold score was defined as predicted positive (P) and a patient with a NEWS2 < threshold score as predicted negative (N). At different NEWS2 threshold values, performance metrics were calculated from 4 different groups: true positive (TP), when an outcome event was correctly predicted, false positive (FP) when an outcome event was predicted but did not occur; with true negative (TN) and false negative (FN) following suit. Receiver operating characteristics (ROC's) were derived from these metrics, for Index-NEWS2 and All-NEWS2.

A range of performance metrics were calculated including two identified to be particularly suited to the representation of early warning score performance, namely the Number Needed to Evaluate (NNE) and the Alert Rate [25]. NNE is the number of patients meeting a threshold NEWS2 score, to include one who then sustains an outcome event, defined as follows:

$$NNE = \frac{TP + FP}{TP} = \frac{1}{PPV}$$

The Alert Rate is the number of threshold scores (alerts that would be generated) per 100 inpatients per day, defined as follows:

$$Alert\ Rate = \frac{TP + FP}{TP + FP + TN + FN}\ x\ number\ of\ NEWS2\ scores\ acquired\ (/100\ patients/day)$$

The NNE and Alert Rate were plotted against the sensitivity as previously discussed [25]. For the Index-NEWS2, an outcome event rate per 100 patient-days, was calculated (Overall Event Rate) as follows:

$$Overall\ Outcome\ Event\ Rate = \frac{TP + FN}{TP + FP + TN + FN}\ x\ 100\ /100\ \text{patient days}$$

For the Index-NEWS2, an outcome event rate at any given threshold score (Alerted Event Rate) per 100 patient-days, was calculated as follows:

$$Alerted\ Outcome\ Event\ Rate = \frac{TP}{TP + FP + TN + FN}\ x\ 100\ /100\ \text{patient days}$$

For the purpose of reporting any daily rate, time of discharge was not considered, a whole day was counted.

Estimation of Clinician Resource Requirement

The clinical resource required to support response recommendations was illustrated by assuming 1 hour of healthcare professional time per clinical evaluation, informed by a report of a rapid clinical response team on a surgical ward in the Netherlands [26]. The number of healthcare professionals required /100 beds occupied was calculated based upon a 40-hour working week and 18% overhead for leave (=1760 hours/year).

Statistical analysis

All statistical analyses were undertaken in STATA SE 15.1. Normally distributed variables are represented as mean +/- standard deviation others as median and interquartile range. Bootstrap analyses of the ROC were undertaken with 10,000 repetitions of 10,000 patient-days. There were no adjustments for multiple comparisons and all p values are reported.

For peer review only - http://bmjopen.bmj.com/site/about/guidelines.xhtml

RESULTS

Patients and admissions

There were 100,362 admissions across 273 days. In 21,744 no NEWS2 was recorded, 21,599 (99.3%) of which were associated with a short length of stay < 12 hours (Figure 1a). The other 78,431 admissions in which NEWS2 was recorded, occurred in 52,214 patients (Table 1).

Table 1 Demographic and clinical characteristics of patients

	Elective	Emergency
Number of patients with one or more included admission eligible for analysis (first admission)	22,538	29,122
Mean age / years (at time of first admission)	53.9 ± 18.1	56.2 ± 21.7
Number of male patients (percentage)	12,142 (53.9%)	13,800 (47.4%)
Ethnicity		
White	16,153 (71.7%)	19,596 (67.3%)
South Asian	2,642 (11.7%)	4,203 (14.4%)
Black	943 (4.2%)	1,489 (5.1%)
Other	806 (3.6%)	1434 (4.9%)
Not Known	1,994 (8.8%)	2,400 (8.2%)
**Admitting speciality on first admission**		
General Medicine	1,545(6.9%)	20,174 (69.3%)
General Surgery	2,150 (9.5%)	3,061 (10.5%)
Trauma & Orthopaedics	2,307 (10.2%)	1,486 (5.1%)
Neurosurgery	1,866 (8.3%)	639 (2.2%)
Urology	1,411 (6.3%)	587 (2.0%)
Cardiology	1,821 (8.1%)	469 (1.6%)
Clinical & Medical Oncology	1,263 (5.6%)	549 (1.9%)
Ear, Nose and Throat (ENT)	1,401 (6.2%)	389 (1.3%)
Plastic Surgery	2,138 (9.5%)	304 (1.0%)
Maxillo-Facial Surgery	849 (3.8%)	252 (0.9%)
All others	5,787 (25.7%)	1,212 (4.2%)
Mean number of admissions / patient	1.6	1.5
Number of admissions in which the patient was ineligible for analysis throughout an admission	60	494

Table 1 Legend

The demographics, mode of admission and admitting speciality on first admission, of the 52,214 patients that were subject to NEWS2 analysis, contributing 36,182 elective and 42,249 emergency admissions. In 554 admissions the patient was ineligible for inclusion in analysis throughout the spell (187 due to admission and discharge from ICU. 367 due to a DNACPR decision).

For peer review only - http://bmjopen.bmj.com/site/about/guidelines.xhtml

DNACPR and outcome

A DNACPR decision was made in 4,621 (4.6%) admissions. This resulted in a DNACPR that was active in 170 ± 15 (13%) in-patients at midday (Table S4 of the online supplement). Of 1076 deaths not on ICU, 943 (87.6%) occurred in those with a DNACPR decision, in 834 of whom this had been in place >24 hours.

Index-NEWS2 recording and outcome

A DNACPR was active throughout the course of 367 admissions. In the remaining 77,877 admissions, 294,602 Index-NEWS2 were recorded. They were associated with 715 outcome events in the following 24 hours (154 deaths and 561 admissions to ICU). Of 10,731 post-admission days in which no NEWS2 was calculated, 5,786 (53.9%) involved discharge later that day, whilst 4,424 (41.2%) comprised a whole day in which the patient was eligible for inclusion and in which no outcome event occurred (See Figure S1 of the online supplement).

Day 1 post-elective admission

Exclusion of Day 1 of elective admission from the analysis of Index-NEWS2 associated outcome, was supported by finding that 94.7% of admissions were followed by a planned procedure, that this occurred within 12 hours, and all outcome events followed a procedure. (The performance of NEWS2 across day 1 post elective admission is shown in Figure S2a of the online supplement)

Classification performance of NEWS2

Across all other days, 1,162,824 All-NEWS2 scores were recorded on 258,678 post-admission days, so a mean of 4.5 NEWS2 were recorded per day. 580/258,678 (0.22%) Index-NEWS2 were associated with an outcome event. 5,284/1,162,824 (0.45%) All-NEWS2 were associated with an outcome event, since a mean of 9 NEWS2 were recorded in the 24 hours prior to an outcome event. The c-statistic of outcome prediction was higher when derived from All-NEWS2 compared to Index-NEWS2 (0.85 vs 0.78; p<0.001; see Figure S2b of the online supplement).

Alert Rate and NNE associated with Index-NEWS2

Table 2 presents Index-NEWS2 performance. The Index-NEWS2 Alert Rate is shown in Table 3. Figure 2a plots this Alert Rate against sensitivity for the Index-NEWS2 score. Around current key clinical response thresholds, the relationship was log-linear: a 2.0 x increase in the Alert Rate for an

For peer review only - http://bmjopen.bmj.com/site/about/guidelines.xhtml

increase in sensitivity of 0.1 (Alert Rate = $0.19e^{7.18x\text{sensitivity}}$ between NEWS2 ≥10 and ≥2). At the key action threshold NEWS2 ≥5 the Alert Rate generated by an Index-NEWS2 = 5.3/100 patients/day, at which score the Index-NEWS2 sensitivity = 0.46.

Figure 2b shows the relationship between the NNE and sensitivity. Around current key clinical response thresholds, the relationship was log-linear: a 1.5 x increase in NNE to increase sensitivity by 0.1 (NNE = $8.7\ e^{4.14x\text{sensitivity}}$ between NEWS2 ≥10 and ≥2). At the key action threshold NEWS2 score ≥5, the NNE = 52.

For peer review only

For peer review only - http://bmjopen.bmj.com/site/about/guidelines.xhtml

Table 2 Performance of Index NEWS 2

Threshold NEWS2	Number of Index NEWS2 meeting threshold	Number of composite outcome events associated with Index NEWS2 at threshold	Alert Rate / 100 patients / post-admission day (95% CI)	Sensitivity (%) (95% CI)	Specificity (%) (95% CI)	PPV (%) (95% CI)	NPV (%) (95% CI)	NNE	Alerted outcome event rate / 100 patient-days	LR+	LR-
**≥1**	184,867	534	71.5 (71.1-71.8)	92.1 (89.6-94.1)	28.6 (28.4-28.8)	0.3 (0.3-0.3)	99.9 (99.9-100)	347	0.21 (0.19-0.22)	1.3	0.28
**≥2**	101,364	453	39.2 (38.9-39.4)	78.1 (74.5-81.4)	60.9 (60.7-61.1)	0.4 (0.4-0.5)	99.9 (99.9-99.9)	224	0.17 (0.16-0.19)	2.0	0.36
**≥3**	53,713	383	20.8 (20.6-20.9)	66.0 (62.0-69.9)	79.3 (79.2-79.5)	0.7 (0.6-0.9)	99.9 (99.9-99.9)	141	0.15 (0.13-0.16)	3.2	0.43
**≥4**	26,484	318	10.2 (10.1-10.4)	54.8 (50.7-58.9)	89.9 (89.7-90.0)	1.2 (1.1-1.3)	99.9 (99.9-99.9)	84	0.12 (0.11-0.14)	5.4	0.50
**≥5 + Single=3**	21,833	288	8.4 (8.3-8.6)	49.7 (45.5-53.8)	91.7 (91.5-91.8)	1.3 (1.2-1.5)	99.9 (99.9-99.9)	76	0.11 (0.10-0.12)	6.0	0.55
**≥5**	13,793	268	5.3 (5.2-5.4)	46.2 (42.1-50.4)	94.8 (94.7-94.8)	1.9 (1.7-2.2)	99.9 (99.9-99.9)	52	0.10 (0.09-0.12)	8.8	0.57
**≥6**	7,252	216	2.8 (2.7-2.9)	37.2 (33.3-41.3)	97.3 (97.2-97.3)	3.0 (2.6-3.4)	99.9 (99.9-99.9)	34	0.08 (0.07-0.10)	13.7	0.65
**≥7**	3,824	153	1.5 (1.4-1.5)	26.4 (22.8-30.2)	98.6 (98.5-98.6)	4.0 (3.4-4.7)	99.8 (99.8-99.8)	25	0.06 (0.05-0.07)	18.5	0.75
**≥8**	2,095	105	0.8 (0.8-0.8)	18.1 (15.1-21.5)	99.2 (99.2-99.3)	5.0 (4.1-6.0)	99.8 (99.8-99.8)	20	0.04 (0.03-0.05)	23.5	0.83
**≥9**	1,158	75	0.4 (0.4-0.5)	12.9 (10.3-15.9)	99.6 (99.6-99.6)	6.5 (5.1-8.1)	99.8 (99.8-99.8)	16	0.03 (0.02-0.04)	30.8	0.87
**≥10**	613	51	0.2 (0.2-0.3)	8.8 (6.6-11.5)	99.8 (99.8-99.8)	8.3 (6.3-10.8)	99.8 (99.8-99.8)	12	0.02 (0.01-0.03)	40.4	0.91

For peer review only

Table 2 legend

The Index NEWS2 score is the first recorded in each consecutive 24-hour period post-admission. 258,678 Index NEWS2 score were analysed from Day 1 to Day 56 of admission, excluding the first day of elective admission. There were 580 outcome events.

The alerted outcome event rate per 100 patient days is the number of outcome events following an Index NEWS2 meeting the threshold NEWS2 score. The overall outcome event rate = 0.22 per 100 patient-days. PPV is positive predictive value. NPV is negative predictive value. NNE is number needed to evaluate. LR+ is positive likelihood ratio. LR- is negative likelihood ratio.

For peer review only

For peer review only - http://bmjopen.bmj.com/site/about/guidelines.xhtml

Alert Rate associated with All-NEWS2

The Alert Rate at a threshold Index-NEWS2 score represents the rate derived from that single score. However, a mean of 4.5 NEWS2 scores were recorded per post-admission day. The Alert Rate derived from All-NEWS2 scores is shown in Table 3. Figure 2a plots the Alert Rate generated by All-NEWS2 against the sensitivity of Index-NEWS2, across different thresholds. Around current clinical response thresholds, the relationship was log-linear: a 1.8 x increase in the Alert Rate for an increase in sensitivity of 0.1 (Alert Rate = $2.12e^{6.14 x sensitivity}$ between NEWS2 ≥10 and ≥2). At the key action threshold NEWS2 score ≥5, the Alert Rate generated by All-NEWS2 = 37.6/100 patients/day.

Alert Rate associated with Highest-NEWS2

The Alert Rate generated by the Highest NEWS 2 score in each post-admission day is also shown in Table 3. Figure 2a plots the Alert Rate generated by the Highest-NEWS2, against the sensitivity of the Index-NEWS2, across different thresholds. Around the current clinical response thresholds, the relationship was log-linear: a 1.8 x increase in the Alert Rate for an increase in sensitivity of 0.1 (Alert Rate = $0.73e^{6.07 x sensitivity}$ between NEWS2 ≥10 and ≥2). At the key action threshold NEWS2 score ≥5, the Alert Rate generated by the Highest-NEWS2 = 12.3/100 patients/ day.

Incremental clinical resource required by changes to NEWS2 threshold

The number of clinicians required to support responses deployed at the different NEWS2 thresholds, is also estimated in Table 3. This is derived from the Alert Rates defined by the Index-NEWS2, All-NEWS2 and the Highest-NEWS2 at each threshold, with 1 hour assigned per clinical response deployed. Thus, at the key action threshold NEWS2 score ≥5, demand for clinician resource was calculated to be respectively 1.1, 7.8 and 2.6 WTE clinicians /100 in-patients.

Calibration of NEWS2

Calibration was not assessed as NEWS2 does not estimate absolute risk [27]. Usefully, integer changes in NEWS2 threshold were associated with approximately equal relative changes in the odds ratio of outcome events, across a wide range of scores (See Figure S3a and S3b of the online supplement).

For peer review only - http://bmjopen.bmj.com/site/about/guidelines.xhtml

Table 3
Alert Rates and clinician resource required to respond at threshold NEWS2

Threshold NEWS2	Index NEWS2 Alert Rate / 100 patients / post-admission day	WTE clinician / 100 occupied beds to respond to Index NEWS2 at threshold	All-NEWS2 Alert Rate / 100 patients / post-admission day	WTE clinician / 100 occupied beds to respond to All NEWS2 at threshold	Highest-NEWS2 Alert Rate / 100 patients / post-admission day	WTE clinician / 100 occupied beds to respond to Highest NEWS2 at threshold
**≥1**	71.5	14.8	335.4	69.6	91.4	19.0
**≥2**	39.2	8.1	202.3	41.9	64.5	13.4
**≥3**	20.8	4.3	117.6	24.4	39.6	8.2
**≥4**	10.4	2.2	65.3	13.6	22.1	4.6
**≥5 or Single=3**	8.4	1.7	52.9	11.0	19.3	4.0
**≥5**	5.3	1.1	37.6	7.8	12.3	2.6
**≥6**	2.8	0.6	22.2	4.6	7.1	1.5
**≥7**	1.5	0.3	12.9	2.7	4.1	0.9
**≥8**	0.8	0.2	7.6	1.6	2.5	0.5
**≥9**	0.4	0.1	4.4	0.9	1.5	0.3
**≥10**	0.2	<0.1	2.4	0.5	0.9	0.2

Table 3 legend

The Alert Rate is the number of NEWS2 scores that reach threshold per 100 patients per post-admission day. NEWS2 score were analysed from Day 1 to Day 56 of admission, excluding the first day of elective admission. Index NEWS2 reports the first NEWS2 recorded in each post-admission day. All-NEWS2 includes all scores recorded in the admission spell. Highest-NEWS2 reports the highest score recorded in each post-admission day. The whole time equivalent (WTE) clinician resource required to service clinical response recommendations at different thresholds, assumed 1 hour per deployed response and an annual clinician workload = 1760 hours. NEWS2 were excluded from analysis in the presence of a DNACPR decision or when the patient was on ICU

For peer review only - http://bmjopen.bmj.com/site/about/guidelines.xhtml

DISCUSSION

In 2019, NHSE required that NEWS2 be used to monitor all adults in acute hospitals [2], extending its scope to include screening for sepsis. This was linked to clinical response recommendations defined by the RCP, including a key threshold for urgent clinical review at NEWS2≥5 [1]. NICE cautioned that when used in this way, there was a risk that NEWS2 based recommendations could result in new demand, which paradoxically might adversely affect overall care delivery [9]. A need for ongoing evaluation was identified. Our analysis was designed to provide a description of how NEWS2 implementation could affect the disposition of clinical resource, across an acute hospital in-patient population, considering the effects of response modifiers such as resuscitation status as well as repeated measurement in those already identified for increased levels of care.

In contrast to other studies, NEWS2 recorded at the time of a DNACPR was excluded from our analysis. This was possible because time stamped records of both were available in the EHR. Previous reports have excluded patients on end-of-life care pathways, however this had to be inferred from an absence of observations in the 24 hours prior to death [27-29]. Precise delineation of these two groups shows that the majority of deaths occurred in patients with a DNACPR in place for more than 24 hours, whereas ICU admissions occurred in patients without a DNACPR. Although this may be unsurprising, it is important when considering the translation of NEWS2 into clinical practice. Compared to previous reports [27-29], there was a low event rate in the large group of patients who were eligible for outcome-based analysis, because the majority of deaths occurred in patients with a DNACPR. A low event rate in this group is one reason why NEWS2 may not perform as well as expected when translated into clinical practice [9, 15]. It is evident in metrics sensitive to the event rate, such as the NNE. This analysis does not imply that NEWS2 is not applicable to patients with a DNACPR, but it does reflect difficulty in interpreting the real-world consequences of recommended thresholds, if these populations are not analysed separately.

Index-NEWS2 was used to assemble single scores from discrete post-admission timeframes, to limit over-representation of scores recorded for clinical indication rather than routine screening. Clinical practice guidelines, including those associated with NEWS2 [1], require increased monitoring of physiological parameters to track those already identified to be at risk of further deterioration. This requires a progressive representation of risk, a different task to screening a population using routine

observations. Our findings are relevant to the latter. The better classification performance of NEWS2 on All-scores compared to Index-scores implies that NEWS2 discriminates better on days in which data acquisition is more frequent [30]. This conclusion is apparently different to the influential report of Jarvis and colleagues, which found that the c-statistic of NEWS and other scoring systems are little affected when using all or single scores per admission [24]. However, their random selection of single scores across an admission would be expected to mirror the distribution of all scores. In contrast, a methodology employing single scores from a timeframe typical of that between routine reviews [31, 32] is less influenced by superior performance on scores obtained during times of high frequency monitoring. Arguably, our analysis of Index-NEWS2 more closely reflects its use as a screening tool in the interval between ward-round based routine assessment, which is typically 24 hours. This difference in performance is another reason why in clinical practice, NEWS2 might not perform as well as expected from the literature [15].

Even at the lower action threshold ≥5, Index-NEWS2 sensitivity was less than 0.5. Other routes to clinical evaluation, such as routine or symptom-based assessment are therefore also likely to play an important role in identifying deteriorating patients for assessment. The resource with which to deliver these assessments must then compete with that demanded by NEWS2 triggered responses. Diversion of resource is one mechanism by which NEWS2 associated response recommendations could adversely affect patient care, particularly if alert rates are high [9].

In our centre, alert rates would be high at current NEWS2 thresholds. This is consistent with data from other NHS acute hospitals [27, 29]. A meaningful diversion of clinical resource away from other routes to evaluation is therefore possible [9]. Equally, alert fatigue could arise, thereby degrading clinical decision making [33, 34]. In response to this, some centres have developed local policies to manage repeated alerting in some situations [35].

Given that responses to All-NEWS2 may be modified in various ways, including prospective decision-making or censoring by ward-staff at the point of care [36], we also defined an Alert Rate based on the Highest-NEWS2 score each day. This is operationally equivalent to allowing one triggered response per person per day. Although it might underestimate the optimal response rate, this provides additional context with which to understand the boundaries of reasonable clinical resource deployment.

For peer review only - http://bmjopen.bmj.com/site/about/guidelines.xhtml

An optimal threshold score would usually be defined by health economic analysis [8, 37]. This is not possible because there is little evidence quantifying survival benefit from EWS triggered responses [8, 10-17], nor is there a consistent account of the cost of current clinical response recommendations. We assigned 1 hour of clinician time per response, to illustrate the potential resource implications of different thresholds. This was based on a single informative study [26] identified by NICE guidance on emergency and acute care [17]. It evaluated a team with critical care competencies, most like that indicated for NEWS2≥7. We chose not to vary this resource attribution further, as our aim was to provide perspective on scale rather than precise definition of cost.

Although a health economic analysis is not possible, description of the Alert Rate and Event Rate provides some insight into the resource consequences of different NEWS2 thresholds, across an acute hospital in-patient population. For example, reducing the NEWS2 threshold from ≥7 to ≥5 would increase the Alert Rate from 4.1 to 12.3 /100 post-admission days, thereby increasing the modelled demand for healthcare professionals from 0.9 to 2.6 whole time equivalents /100 in-patients. This resource estimate is based upon Highest-NEWS2, so may be an under-estimate. The number of Index-NEWS2 triggered responses followed by an event would increase by 0.04/100 patient-days, but 0.12 events /100 patient-days would still not have been predicted. Even as an approximation, these results reveal that small changes in current thresholds can result in demand for clinical resource that could meaningfully impact delivery through other care pathways.

Our analysis of NEWS2 excluded patients when there was a DNACPR decision. This is a diverse group of patients, including those approaching the end of life as well as those in whom significant intervention is considered. Since they form a minority of in-patients, their exclusion from outcome-based evaluation would not alter our conclusions regarding the use of undifferentiated NEWS2 thresholds. Nevertheless, this population warrants separate analysis, in particular those whose resuscitation status changed in the 24 hours prior to death. This may inform an understanding of how different implementations of EWS's are associated with different in-hospital cardiac arrest rates, possibly because they prompt DNACPR decisions differently [38].

Our analysis also excluded Day 1 of elective admission. This is because different cause-effect relationships during that day significantly confound the relationship between physiological

For peer review only - http://bmjopen.bmj.com/site/about/guidelines.xhtml

derangement and outcome. Previous studies have excluded similar cohorts by not including elective admissions [27-29], or day-case admissions [9, 15]. There was however no evidence that inclusion of elective admissions on Day 1 following their return to the ward, would alter an overall assessment of the utility of NEWS2.

A single centre analysis has limitations with respect to generalisation to other populations. Our underlying data are though consistent with reports from other NHS acute hospitals [27, 29]. Furthermore, an editorial response from 2012, employing unpublished data from users of the VitalPac EWS, suggested that RCP recommendations on NEWS (and now NEWS2) would be unsustainable across an entire in-patient population [39]. This can be understood not simply as an issue of capacity but potential jeopardy arising from the redirection of resource [9]. In an acute admissions unit, where event rates are high and resources already targeted, value may be realised from a representation of risk that supports healthcare professionals tracking of patients [3]. As already discussed, this is a different task to the efficient discrimination required of a screening test, applied across the in-patient population, to trigger further clinical evaluation. This distinction is also relevant to recommendations on the identification of sepsis, in which a NEWS2 ≥5 is used to prompt clinical assessment by a senior decision maker [2, 40]. This threshold was developed from the association between outcome and NEWS [41] or qSOFA [27, 28], in those with features of sepsis. NEWS2 ≥5 used as a screening tool for sepsis has not been directly assessed. Our analysis illustrates the potential real-world consequences of such recommendations, including on the distribution of senior clinical resource [9].

In summary, we identify why NEWS2 may not perform as well as expected when screening the in-patient population. This relates not just to moderate classification performance of NEWS2 in this setting (23), but the consequences of high alert rates, including competition for clinical resource [9] and clinicians' attention [33]. There is a particular need to manage multiple alerts in rapid succession [30, 35]. These problems relate in part to the fact that NEWS2 was evolved for paper-based, or stand-alone implementation. In these settings, alert censoring by ward staff is well documented, whether appropriate or not [19, 20]. EHR's automate alerting, thereby generating different problems, associated with high alert-rates. The EHR offers the opportunity to develop more sophisticated scoring systems incorporating a wide range of data, however correspondence with a national, paper-based system would then be lost [30]. Any such development would require careful evaluation using a suitable methodology, such as cluster randomisation [16, 42]. This

For peer review only - http://bmjopen.bmj.com/site/about/guidelines.xhtml

approach has recently demonstrated improved 30-day mortality in patients identified to be at risk of deterioration in Kaiser-Permanente hospitals. An algorithm was used to assess co-morbid conditions and laboratory parameters, as well as physiological parameters [43]. At the chosen response threshold, only 2.8 alerts per 100 patients per day were generated. This was followed by a complex intervention involving remote review by specially trained nurses, stipulated to avoid alert fatigue in hospital staff. It is therefore a significantly different implementation to NEWS2 in England, but one that demonstrates the potential for targeted assessment using an EHR. Indeed, it may be that simple scoring systems are limited in their capacity to confer net benefit across a diverse population, receiving current standards of routine review [31]. Failure to show survival benefit from EWS triggered responses would in that case, not simply reflect limitations in the methodologies used to undertake assessment [15].

In conclusion, there is a risk that currently constituted NEWS2 based response recommendations could adversely impact the overall delivery of care to an in-patient population [9]. The response to multiple alerts requires better definition and ongoing evaluation. As a result, we would not support undifferentiated implementation of current recommendations at a key threshold NEWS2 score ≥5, across the entire in-patient population at our centre. Given existing reports of NEWS2 performance, our findings are likely to be relevant to other acute hospitals.

For peer review only

DATA SHARING

The anonymised participant data and a data dictionary defining each field will be available to others through application to PIONEER, the HDR-UK Health data Hub via the corresponding author. The data will be available upon request and following approval of a process to ensure ethical data governance and through a data access agreement. Please contact the corresponding author for details.

CONTRIBUTING STATEMENT

Pankhurst, Gkoutos and Ball designed the study. Gyves, Evison, Gallier curated the health data and conducted the analysis. Sapey and Ball wrote the paper. Pankhurst, Gallier, Gkoutos contributed to manuscript revision. All authors approved the final version.

COPYRIGHT AND LICENSE FOR PUBLICATION

The Corresponding Author has the right to grant on behalf of all authors and does grant on behalf of all authors, a worldwide licence to the Publishers and its licensees in perpetuity, in all forms, formats and media (whether known now or created in the future), to i) publish, reproduce, distribute, display and store the Contribution, ii) translate the Contribution into other languages, create adaptations, reprints, include within collections and create summaries, extracts and/or, abstracts of the Contribution, iii) create any other derivative work(s) based on the Contribution, iv) to exploit all subsidiary rights in the Contribution, v) the inclusion of electronic links from the Contribution to third party material where-ever it may be located; and, vi) licence any third party to do any or all of the above.

PATIENT AND PUBLIC INVOLVEMENT

302 patients and public members were consulted in relation to the use of health data to improve the care for people with acute, unplanned illness. The theme of acuity scores was discussed in a working group and agreed to be a priority for patients. The results of this paper will be disseminated through the PIONEER patient and public group.

CONFLICTS OF INTEREST

For peer review only

TP, HG, FE, SG report no conflicts of interest. SB and GG report grant funding from HDR-UK during the conduct of the study. GG further acknowledges the support of the NIHR Birmingham ECMC, the NIHR Birmingham SRMRC and the Nanocommons H2020-EU (731032). ES reports grants from HDR-UK, during the conduct of the study; grants from Medical Research Council, grants from NIHR, grants from Wellcome Trust, grants from British Lung Foundation, grants from Alpha 1 Foundation, outside the submitted work.

For peer review only

For peer review only - http://bmjopen.bmj.com/site/about/guidelines.xhtml

REFERENCES

1. Royal College of Physicians. National Early Warning Score (NEWS) 2: Standardising the assessment of acute-illness severity in the NHS. Updated report of a working party. London: RCP, 2017.
2. NHS England, Royal College of Physicians, NHS Improvement. Patient safety alert: Resources to support the safe adoption of the revised National Early Warning Score (NEWS2). NHS, 2018.
3. Smith GB, Prytherch DR, Meredith P, et al. The ability of the National Early Warning Score (NEWS) to discriminate patients at risk of early cardiac arrest, unanticipated intensive care unit admission, and death. *Resuscitation*. 2013;84(4):465-70.
4. Alam N, Vegting IL, Houben E, et al. Exploring the performance of the National Early Warning Score (NEWS) in a European emergency department. *Resuscitation*. 2015;90:111-5.
5. Abbott TE, Vaid N, Ip D, et al. A single-centre observational cohort study of admission National Early Warning Score (NEWS). *Resuscitation*. 2015;92:89-93.
6. Kovacs C, Jarvis SW, Prytherch DR, et al. Comparison of the National Early Warning Score in non-elective medical and surgical patients. *Br J Surg*. 2016;103(10):1385-93.
7. Mellhammar L, Linder A, Tverring J, et al. Scores for sepsis detection and risk stratification - construction of a novel score using a statistical approach and validation of RETTS. *PLoS One*. 2020;15(2):e0229210.
8. Wuytack F, Meskell P, Conway A, et al. The effectiveness of physiologically based early warning or track and trigger systems after triage in adult patients presenting to emergency departments: a systematic review. *BMC Emerg Med*. 2017;17(1):38.
9. 2019 exceptional surveillance of sepsis: recognition, diagnosis and early management (NICE guideline NG51) and acutely ill adults in hospital: recognising and responding to deterioration (NICE guideline CG50). London: National Institute for Health and Care Excellence (UK) Copyright © NICE 2019.; 2019.
10. Alam N, Hobbelink EL, van Tienhoven AJ, et al. The impact of the use of the Early Warning Score (EWS) on patient outcomes: a systematic review. *Resuscitation*. 2014;85(5):587-94.
11. Schmidt PE, Meredith P, Prytherch DR, et al. Impact of introducing an electronic physiological surveillance system on hospital mortality. *BMJ Quality & Safety*. 2015;24(1):10-20.
12. van Schalkwyk JM. Confounding explains 'deaths avoided'. *BMJ Quality & Safety*. 2015;24(2):175-.
13. Gao H, Harrison DA, Parry GJ, et al. The impact of the introduction of critical care outreach services in England: a multicentre interrupted time-series analysis. *Crit Care*. 2007;11(5):R113.
14. McNeill G, Bryden D. Do either early warning systems or emergency response teams improve hospital patient survival? A systematic review. *Resuscitation*. 2013;84(12):1652-67.
15. Gerry S, Bonnici T, Birks J, et al. Early warning scores for detecting deterioration in adult hospital patients: systematic review and critical appraisal of methodology. *BMJ*. 2020;369:m1501.

For peer review only - http://bmjopen.bmj.com/site/about/guidelines.xhtml

16. Haegdorens F, Van Bogaert P, Roelant E, et al. The introduction of a rapid response system in acute hospitals: A pragmatic stepped wedge cluster randomised controlled trial. *Resuscitation*. 2018;129:127-34.
17. Critical care outreach teams. Emergency and acute medical care in over 16s: service delivery and organisation: NICE; 2018.
18. National Early Warning Score systems that alert to deteriorating adult patients in hospital (MIB 205). Medtech innovation briefing.: NICE; 2020.
19. Credland N, Dyson J, Johnson MJ. What are the patterns of compliance with Early Warning Track and Trigger Tools: A narrative review. *Appl Nurs Res*. 2018;44:39-47.
20. Hope J, Griffiths P, Schmidt PE, et al. Impact of using data from electronic protocols in nursing performance management: A qualitative interview study. *J Nurs Manag*. 2019;27(8):1682-90.
21. Managing Medical Devices. Medicines and Healthcare products Regulatory Agency 2021.
22. Paterson R, MacLeod DC, Thetford D, et al. Prediction of in-hospital mortality and length of stay using an early warning scoring system: clinical audit. *Clin Med (Lond)*. 2006;6(3):281-4.
23. https://datadictionary.nhs.uk/attributes/critical_care_admission_type.html [
24. Jarvis SW, Kovacs C, Briggs J, et al. Are observation selection methods important when comparing early warning score performance? *Resuscitation*. 2015;90:1-6.
25. Romero-Brufau S, Huddleston JM, Escobar GJ, et al. Why the C-statistic is not informative to evaluate early warning scores and what metrics to use. *Crit Care*. 2015;19:285.
26. Simmes F, Schoonhoven L, Mintjes J, et al. Financial consequences of the implementation of a rapid response system on a surgical ward. *J Eval Clin Pract*. 2014;20(4):342-7.
27. Pimentel MAF, Redfern OC, Gerry S, et al. A comparison of the ability of the National Early Warning Score and the National Early Warning Score 2 to identify patients at risk of in-hospital mortality: A multi-centre database study. *Resuscitation*. 2019;134:147-56.
28. Shamout F, Zhu T, Clifton L, et al. Early warning score adjusted for age to predict the composite outcome of mortality, cardiac arrest or unplanned intensive care unit admission using observational vital-sign data: a multicentre development and validation. *BMJ Open*. 2019;9(11):e033301.
29. Smith GB, Prytherch DR, Jarvis S, et al. A Comparison of the Ability of the Physiologic Components of Medical Emergency Team Criteria and the U.K. National Early Warning Score to Discriminate Patients at Risk of a Range of Adverse Clinical Outcomes. *Crit Care Med*. 2016;44(12):2171-81.
30. Zhu Y, Chiu YD, Villar SS, et al. Dynamic individual vital sign trajectory early warning score (DyniEWS) versus snapshot national early warning score (NEWS) for predicting postoperative deterioration. *Resuscitation*. 2020;157:176-84.
31. NHS Engalnd Seven Day Services Standards. 2017.
32. Kipnis P, Turk BJ, Wulf DA, et al. Development and validation of an electronic medical record-based alert score for detection of inpatient deterioration outside the ICU. *J Biomed Inform*. 2016;64:10-9.

For peer review only

33. Coleman JJ, van der Sijs H, Haefeli WE, et al. On the alert: future priorities for alerts in clinical decision support for computerized physician order entry identified from a European workshop. *BMC Med Inform Decis Mak*. 2013;13:111.
34. Bedoya AD, Clement ME, Phelan M, et al. Minimal Impact of Implemented Early Warning Score and Best Practice Alert for Patient Deterioration. *Crit Care Med*. 2019;47(1):49-55.
35. Redhead J. Personal communication. Time protocols for muting sepsis alerts when a clinical assessment indicates that a patient does not have sepsis but continues to have a high NEWS score. Results of a survey undertaken on behalf of the Shelford Group Medical Directors. 2019.
36. Escobar GJ, LaGuardia JC, Turk BJ, et al. Early detection of impending physiologic deterioration among patients who are not in intensive care: development of predictive models using data from an automated electronic medical record. *J Hosp Med*. 2012;7(5):388-95.
37. Murphy A, Cronin J, Whelan R, et al. Economics of Early Warning Scores for identifying clinical deterioration-a systematic review. *Ir J Med Sci*. 2018;187(1):193-205.
38. Hogan H, Hutchings A, Wulff J, et al. Interventions to reduce mortality from in-hospital cardiac arrest: a mixed-methods study. 2019;7:2.
39. McGinley A, Pearse RM. A national early warning score for acutely ill patients. *BMJ : British Medical Journal*. 2012;345:e5310.
40. Sepsis guidance implementation advice for adults. NHS England. London; 2017.
41. Corfield AR, Lees F, Zealley I, et al. Utility of a single early warning score in patients with sepsis in the emergency department. *Emerg Med J*. 2014;31(6):482-7.
42. Bonnici T, Gerry S, Wong D, et al. Evaluation of the effects of implementing an electronic early warning score system: protocol for a stepped wedge study. *BMC Medical Informatics and Decision Making*. 2016;16(1):19.
43. Escobar GJ, Liu VX, Schuler A, et al. Automated Identification of Adults at Risk for In-Hospital Clinical Deterioration. *N Engl J Med*. 2020;383(20):1951-60.

For peer review only

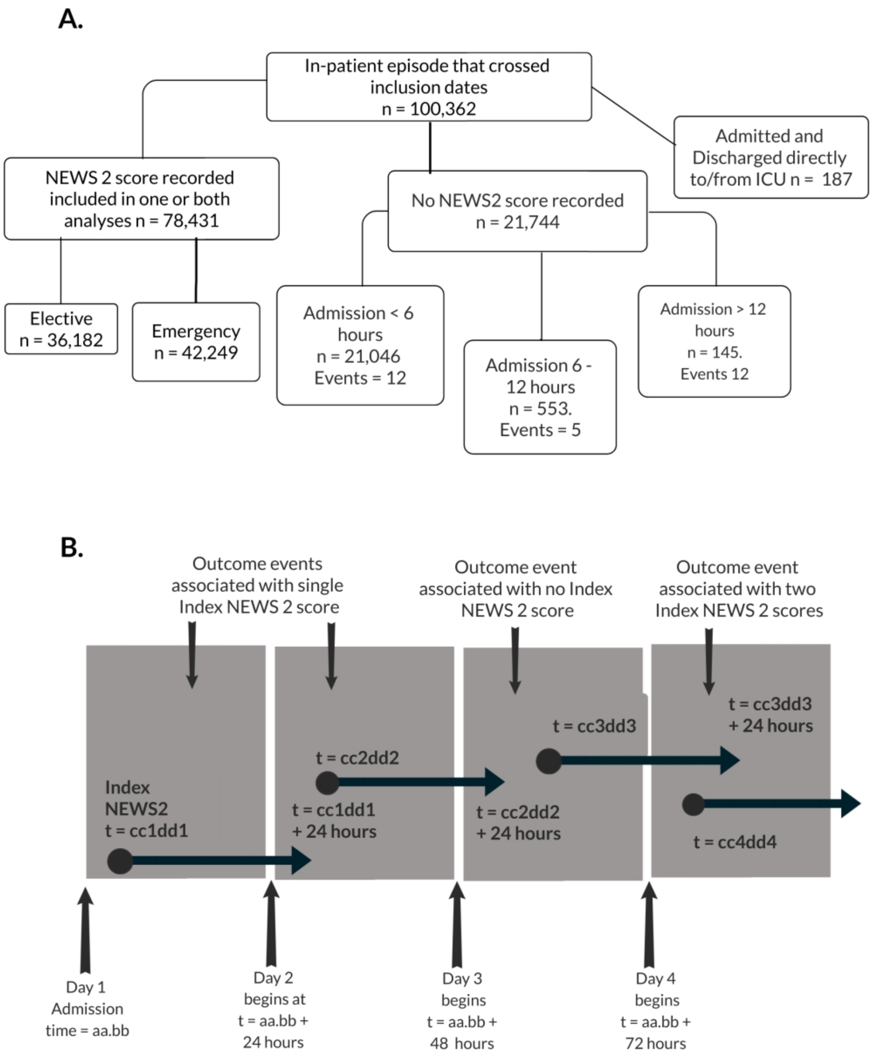

Figure 1 Modified consort diagram and overview of study design

Legend

a. Of 100,762 consecutive in-patient spells, 78,431 (77.8%) recorded at least one NEWS2 score whilst admitted outside of ICU. Of the remaining 21,931, 0.8% were admitted and discharged from ICU and so were never eligible for inclusion in the analysis. Of the others in which no NEWS2 score was recorded 96.8% had a length of stay (LOS) < 6 hours, 99.3% <12 hours (and 0.3% ≥24 hours). There were 29 deaths or admission to ICU associated with spells in which no NEWS2 was recorded.

b. Post-admission days were defined as consecutive 24-hour periods beginning at the time of admission T. The Index NEWS2 score was the first recorded in each of these periods at time t (black circle). A distinct overlapping 'patient-day' variable was then defined for each NEWS2 score between t and t + 24 hours during which, occurrence of a composite outcome event (black diamond) was recorded and linked to the preceeding Index NEWS2. Since the exact time t , that the Index-NEWS2 was recorded varies on each day, a small number of outcome events may be associated with no Index NEWS2 score (3.5%) as illustrated during Day 3, or with two Index NEWS2 scores (9.6%) as illustrated during Day 4.

831x1076mm (72 x 72 DPI)

Figure 2A

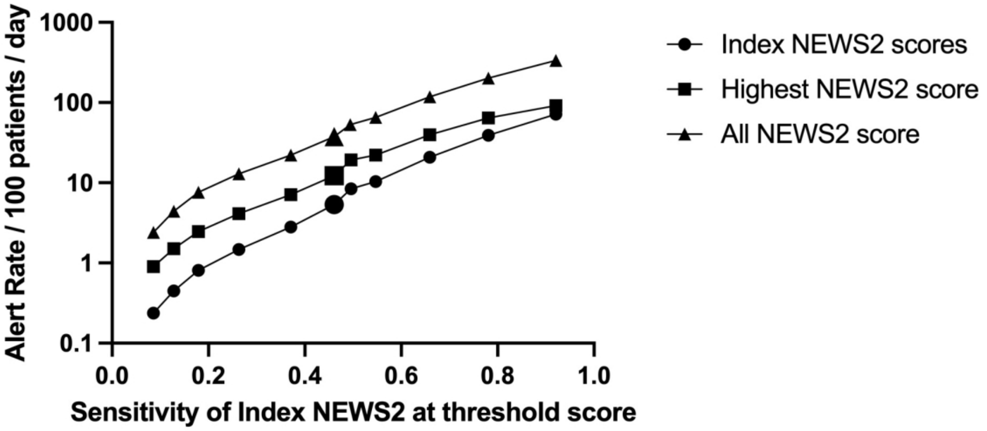

Figure 2B

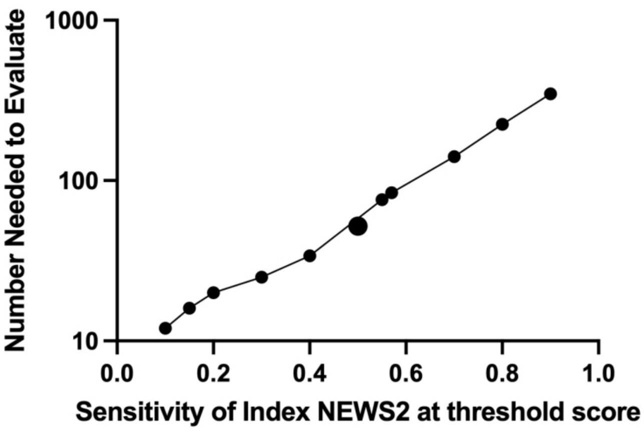

Figure 2 Alert Rate and Number Needed to evaluate for NEWS2

Legend

a. Alert Rate for Index, All and Highest NEWS2 score vs sensitivity for Index NEWS2 score

The Alert Rate is the number of NEWS2 scores recorded at any given threshold, per 100 patients per post-admission day.

NEWS2 scores from Day 1 to 56 post-admission other than for Day 1 of elective admission were evaluated. The larger marker denotes the key action threshold NEWS2 = or > 5, to the right of which is the marker for NEWS2 =or >5 or single parameter = 3 (and then NEWS2 = or > 4, = or > 3, = or >2, = or >1), and to left of which is NEWS2 = or > 6 (and then NEWS2 = or >7, = or >8, = or >9, = or >10).

b. Number Needed to Evaluate vs sensitivity for Index NEWS2 score

The Number Needed to Evaluate is the number of patients to whom an Index NEWS2 score based clinical response must be deployed at threshold, to include one who then sustains a linked composite outcome event. The composite outcome event was defined as the first of unplanned admission to ICU (type 1 and 2 of the NHS critical care minimum dataset) or death of the patient within 24 hours of a NEWS2 score.

158x175mm (300 x 300 DPI)

An Evaluation of NEWS2 response thresholds in a retrospective observational study from a UK acute hospital

Online supplement.

Dr Tanya Pankhurst, Dr Elizabeth Sapey, Ms Helen Gyves, Ms Felicity Evison, Ms Suzy Gallier, Professor Georgios Gkoutos, Professor Simon Ball

For peer review only

Online Supplementary Figure 1 (Figure S1)

Post admission days with no Index NEWS2 recorded

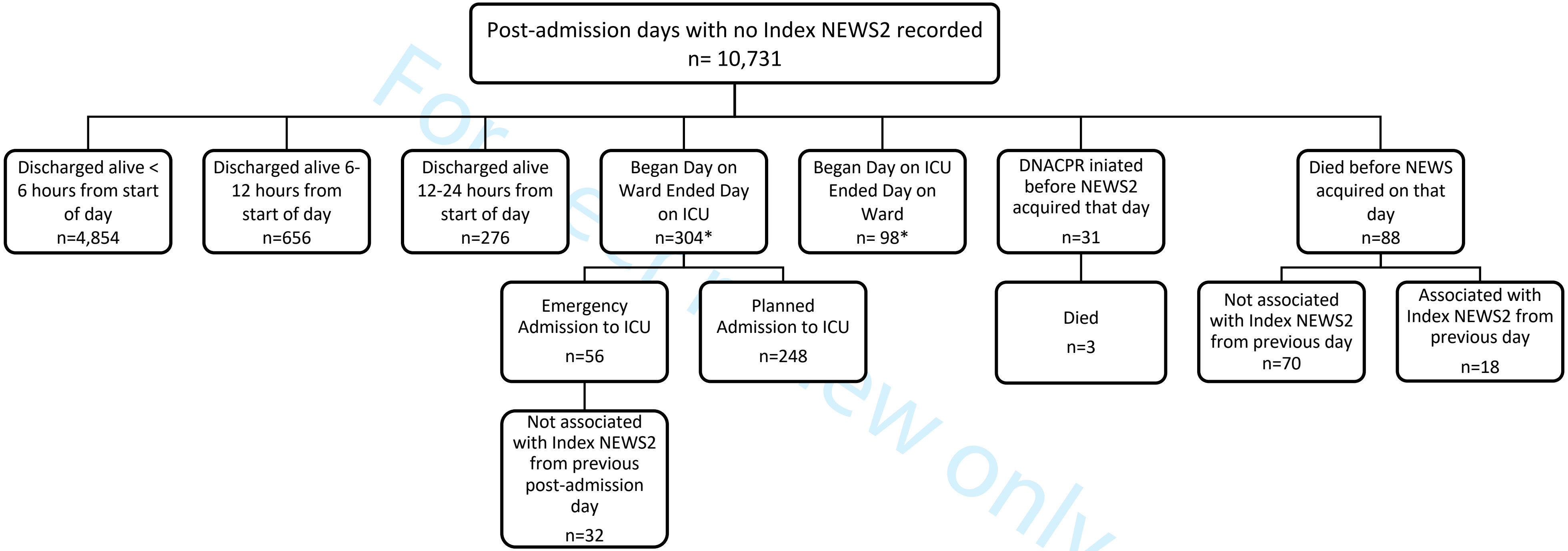

Online Supplementary Figure 1 (Fig S1)

Post admission days with no Index NEWS2 recorded

Of the 10,731 in which no Index-NEWS2 was recorded, 5,786 (53.9%) were discharged alive later that day. 433 (4.0%) were ineligible for part of the day because they were on ICU or had a DNACPR initiated. A further 88 (0.8%) died before a NEWS2 was recorded on that day. 4,424 post-admission days were therefore not associated with a NEWS2 record for unidentified reasons.

There were 144 events (88 deaths and 56 emergency admissions to ICU) associated with post-admission days in which no Index-NEWS2 was recorded and another 3 in which a DNACPR was initiated prior to NEWS2 acquisition and subsequent death. Of the 144 events, 102 were not associated with an Index NEWS2 from the previous day, 20 because they occurred on the first day. The other 82 were associated with NEWS2 acquired later in the previous day but occurred > 24 hours after that day's Index-NEWS2.

For peer review only

Online Supplementary Figure 2a (Fig S2a)

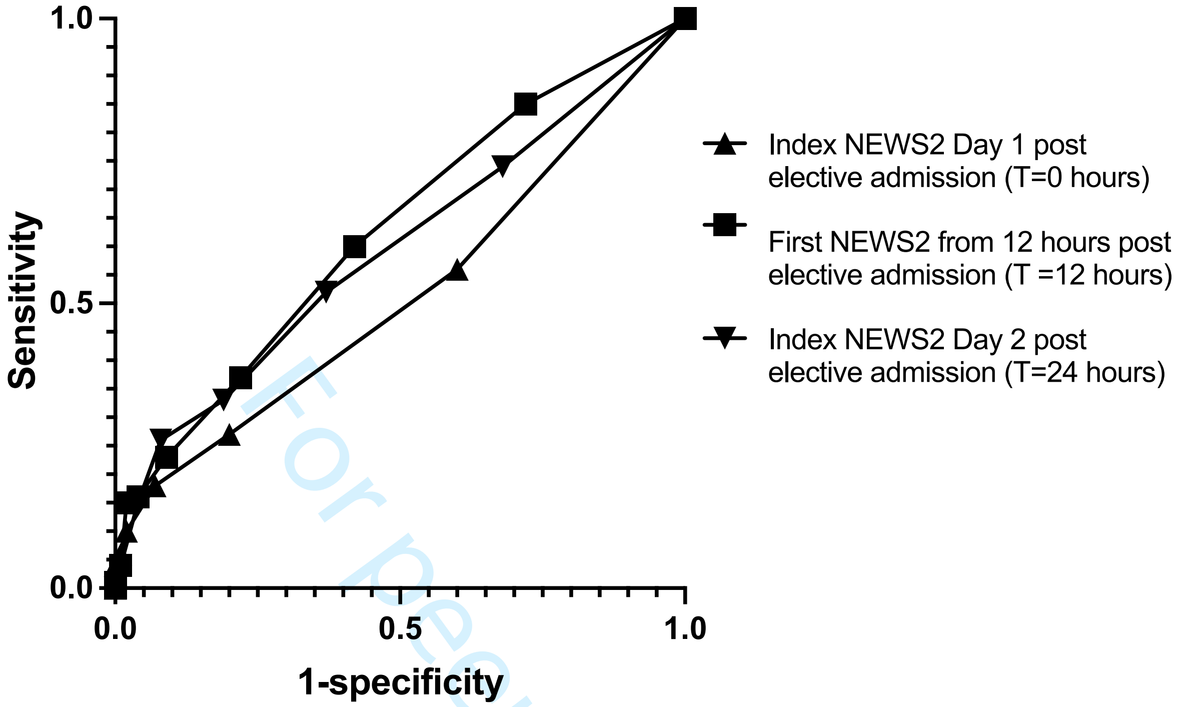

Legend Figure S2a

Receiver operating characteristic (ROC) for the first NEWS2 obtained from 0-, 12- and 24-hours post elective-admission. The respective c-statistics were 0.51, 0.63 and 0.60. The c-statistic = 0.74 for all later post elective admission patient-days combined. The outcome event was the first of unplanned admission to ICU (type 1 and 2 of the NHS critical care minimum dataset) or death of the patient within 24 hours of an evaluated NEWS2 score. As anticipated in the study design, Index NEWS2 on Day 1 of elective admission was not predictive of an adverse outcome. Its acquisition almost always preceded physiological insult, specifically a planned procedure occurring in 94.7% of patients in the subsequent 12 hours. Assessment of the discriminatory power of NEWS2 in the hours immediately following a procedure is beyond the scope of this analysis but appears low.

The NEWS2 associated event rate on Day 1 post elective-admission = 0.37/100 patient-days and Day 2 = 0.34/100 patient-days.

Online Supplementary Figure 2b (Fig S2b)

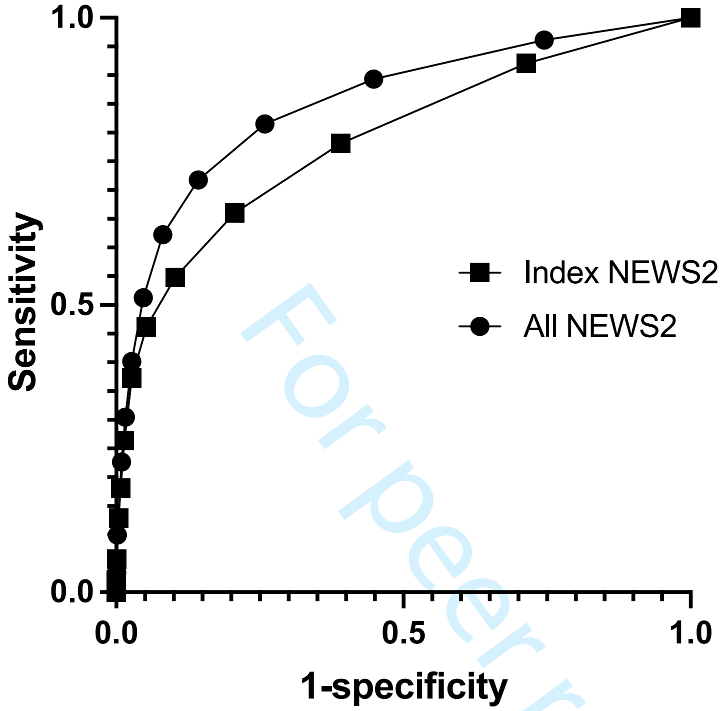

Legend Figure S2b

Receiver operating characteristic (ROC) for Index NEWS2 recorded in Day 1 to 56 excluding first post elective admission day (c statistic = 0.78, 95% CI:0.76-0.81) and All NEWS2 recorded in Days 1 to 56 excluding first post elective admission day (c statistic = 0.85, 95% CI: 0.85-0.86) $p<0.001$. A bootstrap analysis was performed with 10,000 repetitions of 10,000 patient-days, the median and 2.5th and 97.5th percentiles were then used to generate the ROC curve and corresponding confidence intervals. The outcome event was defined as the first of unplanned admission to ICU (type 1 and 2 of the NHS critical care minimum dataset) or death of the patient within 24 hours of a NEWS2 score.

For peer review only

Online Supplementary Figure 3a (Fig S3a)

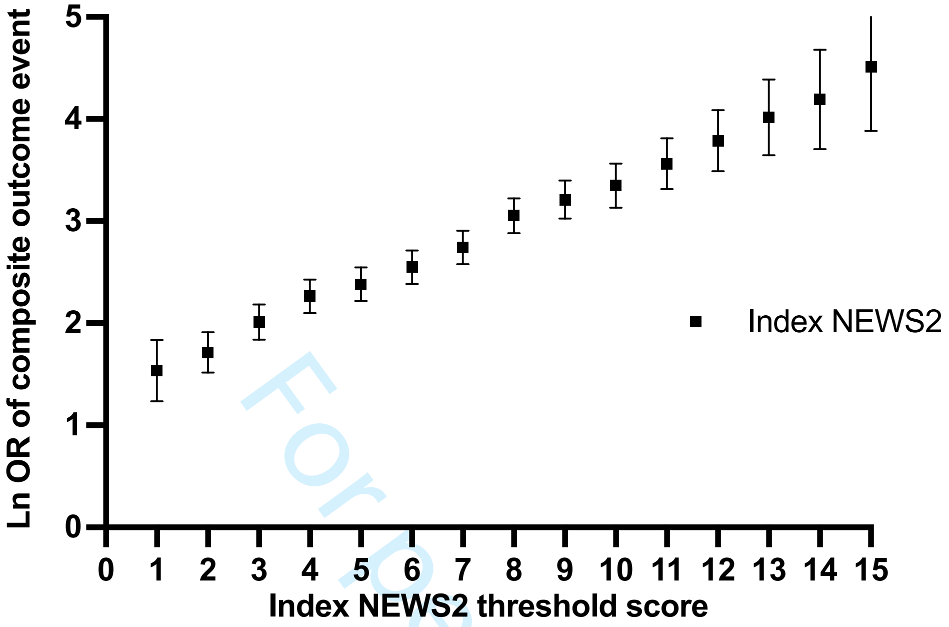

Legend Figure S3a

The Ln of the Odds Ratio (Ln OR) ± 95% confidence intervals for occurrence of the composite outcome event when the Index NEWS2 ≥ threshold score *vs.* < threshold score. The composite outcome event was the first of unplanned admission to ICU (type 1 and 2 of the NHS critical care minimum dataset) or death of the patient within 24 hours of a NEWS2 score. This analysis included NEWS2 scores from Day 1 to 56 post-admission other than for Day 1 of elective admission (Table S2a and S2b).

Although NEWS2 is not constituted to report absolute risk, it exhibits features useful in representing risk. Integer changes in threshold were associated with an approximately equal change in the odds ratio of an outcome event. Across the range of scores, the OR of an event ≥ threshold score *vs.* < threshold score, increased by a factor of 1.2. (Ln OR of composite outcome event = 1.35 + 0.205 (Index NEWS2 threshold score); R^2 = 0.99. For increase in NEWS2 =1, the increase in Ln OR of composite outcome event = 0.205, the OR of composite outcome event increases by a factor of $e^{0.205}$ = 1.2).

(These relationships resemble Fig 2b, because the relationship between sensitivity and Index NEWS2 is approximately negative linear and because at low event rates the odds of an event at threshold ≈ PPV = 1/NNE; the Ln (odds of an event) ≈ -Ln (NNE)).

Online Supplementary Figure 3b (Fig S3b)

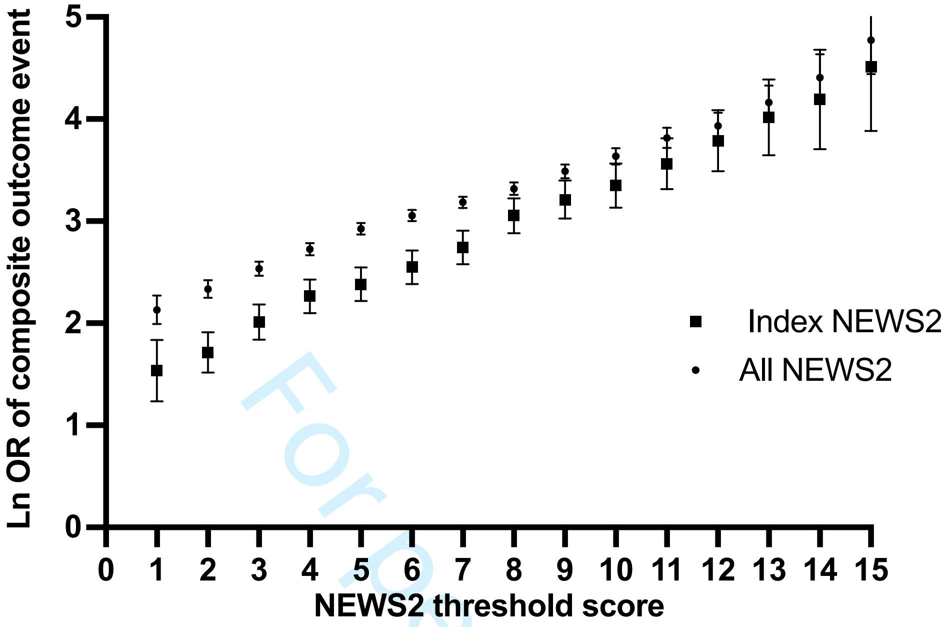

Legend Figure S3b

The Ln of the Odds Ratio (Ln OR) ± 95% confidence intervals for occurrence of the composite outcome event when the Index NEWS2 or All NEWS2 ≥ threshold score *vs.* < threshold score. This representation of the data reflects the differences in performance between Index-and All-NEWS2 shown in Fig S2b.

For peer review only

Supplementary Table S1 NEWS2 and SEWS scoring

National Early Warning Score (NEWS) 2 Standardising the assessment of acute-illness severity in the NHS– Royal College Physicians, London

Chart1: The NEWS2 scoring system

**Physiological parameter**				**Score**			
	3	2	1	0	1	2	3
Respiration rate (per minute)	≤8		9–11	12–20		21–24	≥25
SpO_2 Scale 1 (%)	≤91	92–93	94–95	≥96			
SpO_2 Scale 2 (%)	≤83	84–85	86–87	88–92 ≥93 on air	93–94 on oxygen	95–96 on oxygen	≥97 on oxygen
Air or oxygen?		Oxygen		Air			
Systolic blood pressure (mmHg)	≤90	91–100	101–110	111–219			≥220
Pulse (per minute)	≤40		41–50	51–90	91–110	111–130	≥131
Consciousness				Alert			CVPU
Temperature (°C)	≤35.0		35.1-36.0	36.1-38.0	38.1-39.0	≥39.1	

Chart 2: NEWS2 thresholds and triggers

**NEW score Clinical risk Response**		
Aggregate score 0–4	Low	Ward-based response
Red score<br>Score of 3 in any individual parameter	Low–medium	Urgent ward-based response*
Aggregate score 5–6	Medium	Key threshold for urgent response*
Aggregate score 7 or more	High	Urgent or emergency response**

* Response by a clinician or team with competence in the assessment and treatment of acutely ill patients and in recognising when the escalation of care to a critical care team is appropriate.
**The response team must also include staff with critical care skills, including airway management.
National Early Warning Score (NEWS) 2 Standardising the assessment of acute-illness severity in the NHS– Royal College Physicians, London,
© Royal College of Physicians 2017

Standardised early warning scoring system (SEWS) parameters and scoring system.

**Physiological parameter**				**Score**			
	3	2	1	0	1	2	3
Respiration rate (per minute)	≤8			9–20	21-30	31-35	≥36
SaO_2	<85	85-89	90-92	≥93			
Temperature (°C)	≤33.9	34.0-34.9	35.0-35.9	36.0-37.9	38.0-38.9	≥39.0	
Blood pressure (mmHg)	≤69	70-79	80-99	100-199		≥200	
Pulse (per minute)	≤29	30-39	40-49	50-99	100-109	110-129	≥130
AVPU				Alert	Verbal	Pain	None

Supplementary Table S2 – Index NEWS2 and SEWS (a) and linked outcomes events (b) from Day 1 to 56 post-admission excluding Day 1 of elective admission

Table S2a Index NEWS2 and SEWS

		NEWS2																				
		0	1	2	3	4	3 single	4 single	5	6	7	8	9	10	11	12	13	14	15	16	17	Total SEWS
	0	72852	54298	21005	6960	2310	123	110	891	232	40	5	1	1	0	0	0	0	0	0	0	158828
	1	674	28195	22333	10054	3685	2628	1565	2212	867	292	57	19	3	1	1	0	0	0	0	0	72586
	2	283	835	3995	4771	2162	1029	1512	1910	952	427	174	60	16	3	1	0	0	0	0	0	18130
	3	2	170	291	800	925	445	478	1068	793	481	246	102	35	17	4	0	0	0	0	0	5857
	4	0	5	27	43	156	9	123	394	431	301	211	143	68	15	7	2	2	0	0	0	1937
SEWS	5	0	0	0	4	10	3	14	62	129	143	157	118	85	39	16	4	6	0	1	0	791
	6	0	0	0	0	0	0	0	5	21	40	66	66	59	46	23	10	2	0	0	0	338
	7	0	0	0	0	0	0	0	0	1	5	15	27	24	26	12	8	2	2	1	0	123
	8	0	0	0	0	0	0	0	0	2	0	6	7	7	10	10	5	4	3	1	1	56
	9	0	0	0	0	0	0	0	0	0	0	0	2	2	2	4	4	2	2	0	0	18
	10	0	0	0	0	0	0	0	0	0	0	0	0	0	2	1	2	1	0	1	2	9
	11	0	0	0	0	0	0	0	0	0	0	0	0	0	1	0	1	0	0	1	0	3
	12	0	0	0	0	0	0	0	0	0	0	0	0	0	0	0	0	0	1	0	0	1
	13	0	0	0	0	0	0	0	0	0	0	0	0	0	0	0	0	0	0	0	1	1
	Total	73811	83503	47651	22632	9248	4237	3802	6542	3428	1729	937	545	300	162	79	36	19	8	5	4	
	Cum Total	73765	157187	204768	231572	244572			251062	254427	256108	257015	257536	257818	257966	258038	258068	258083	258090	258094	258098	

Of the 8039 that NEWS2 = 3 in any single parameter 149 (1.8%) triggered the lowest SEWS threshold ≥4
Of the 6542 that NEWS2 = 5, 461 triggered (7.0%) the lowest SEWS threshold ≥4
Of the 3428 that NEWS2 = 6, 584 triggered (16.0%) the lowest SEWS threshold ≥4
Cum Total Is the cumulative total at or below the NEWS2 score *without an event* used to calculate the odds ratio of the composite outcome event when the Index NEWS2 ≥ threshold score *vs.* < threshold score (Fig S3a).

For peer review only

Table S2b outcome events linked to Index NEWS2 and SEWS

		NEWS2																				
		0	1	2	3	4	3 single	4 single	5	6	7	8	9	10	11	12	13	14	15	16	17	Total SEWS
SEWS	0	45	52	28	21	9	0	0	3	2	1	0	0	0	0	0	0	0	0	0	0	161
	1	0	25	30	19	19	5	3	12	18	5	0	1	0	0	0	0	0	0	0	0	137
	2	1	3	11	11	7	2	5	20	16	6	5	3	1	0	1	0	0	0	0	0	92
	3	0	1	1	4	5	3	1	14	16	20	8	7	0	0	1	0	0	0	0	0	81
	4	0	0	0	0	0	0	1	3	8	7	5	4	1	1	0	0	0	0	0	0	30
	5	0	0	0	0	0	0	0	0	2	8	9	6	8	4	3	0	2	0	0	0	42
	6	0	0	0	0	0	0	0	0	1	1	3	1	6	4	2	1	0	0	0	0	19
	7	0	0	0	0	0	0	0	0	0	0	0	0	2	5	0	2	0	0	0	0	9
	8	0	0	0	0	0	0	0	0	0	0	0	2	0	0	0	1	2	1	1	0	7
	9	0	0	0	0	0	0	0	0	0	0	0	0	0	0	0	1	0	0	0	0	1
	10	0	0	0	0	0	0	0	0	0	0	0	0	0	0	0	1	0	0	0	0	1
	11	0	0	0	0	0	0	0	0	0	0	0	0	0	0	0	0	0	0	0	0	0
	12	0	0	0	0	0	0	0	0	0	0	0	0	0	0	0	0	0	0	0	0	0
	13	0	0	0	0	0	0	0	0	0	0	0	0	0	0	0	0	0	0	0	0	0
	Total	46	81	70	55	40	10	10	52	63	48	30	24	18	14	7	6	4	1	1	0	
	Cum Total	46	127	197	262	312			364	427	475	505	529	547	561	568	574	578	579	580	580	

Of the 20 that NEWS2 = 3 in any single parameter, 1 (5.0%) triggered the lowest SEWS threshold ≥4
Of the 52 that NEWS2 = 5, 3 (5.8%) the lowest SEWS threshold ≥4
Of the 63 that NEWS2 = 6, 11 triggered (17.5%) the lowest SEWS threshold ≥4
Cum Total Is the cumulative total at or below the NEWS2 score *with an event* used to calculate the odds ratio of the composite outcome event when the Index NEWS2 ≥ threshold score *vs.* < threshold score (Fig S3a).

For peer review only

Supplementary Table S3 – All NEWS2 and SEWS (a) and linked outcomes events (b) from Day 1 to 56 post-admission excluding Day 1 of elective admission

Table S3a All NEWS2 and SEWS

		NEWS2																					
		0	1	2	3	4	3 single	4 single	5	6	7	8	9	10	11	12	13	14	15	16	17	18	Total SEWS
SEWS	0	291135	225784	99131	37585	12886	631	500	4720	1444	269	46	11	1	0	0	0	0	0	0	0	0	674143
	1	2748	114393	99833	49029	20759	12435	7958	13629	6182	2206	696	143	34	5	1	0	0	0	0	0	0	330051
	2	1159	3772	18598	24228	12226	4894	7661	11932	7365	3669	1681	666	175	37	11	1	0	0	0	0	0	98075
	3	10	583	1364	3940	5404	2053	2820	6782	5438	3983	2413	1231	462	188	54	12	2	0	0	0	0	36739
	4	0	15	113	279	778	55	653	2462	2560	2349	1883	1428	768	302	122	33	9	3	0	0	0	13812
	5	0	0	2	19	53	11	44	369	809	1021	1044	1024	763	442	184	76	28	9	1	0	0	5899
	6	0	0	0	0	2	0	3	15	105	227	365	503	485	368	213	112	48	12	3	0	0	2461
	7	0	0	0	0	0	0	0	1	13	27	83	166	158	197	151	89	59	17	8	0	2	971
	8	0	0	0	0	0	0	0	0	3	4	19	30	62	98	64	64	40	22	9	2	1	418
	9	0	0	0	0	0	0	0	0	0	0	3	5	13	19	40	35	22	20	11	3	0	171
	10	0	0	0	0	0	0	0	0	0	0	0	2	2	4	11	10	12	6	9	4	1	61
	11	0	0	0	0	0	0	0	0	0	0	0	0	0	1	1	3	2	2	4	0	0	13
	12	0	0	0	0	0	0	0	0	0	0	0	0	0	0	0	1	3	2	1	1	0	8
	13	0	0	0	0	0	0	0	0	0	0	0	0	0	0	0	0	0	1	0	1	0	2
	Total	295052	344547	219041	115080	52108	20079	19639	39910	23919	13755	8233	5209	2923	1661	852	436	225	94	46	11	4	1,162,824
	Cum Total	294846	639036	857663	992306	1063551			1102881	1126214	1139456	1147276	1152092	1154736	1156173	1156904	1157262	1157439	1157501	1157530	1157539	1157540	

Of the 39178 that NEWS2 = 3 in any single parameter, (1.9%) 766 triggered the lowest SEWS threshold ≥4
Of the 39910 that NEWS2 = 5, 2847 triggered (7.1%) the lowest SEWS threshold ≥4
Of the 23919 that NEWS2 = 6, 3490 triggered (14.5%) the lowest SEWS threshold ≥4
Cum Total Is the cumulative total at or below the NEWS2 score *without an event* used to calculate the odds ratio of the composite outcome event when the Index NEWS2 ≥ threshold score *vs.* < threshold score (Fig S3b).

For peer review only

Table S3b outcome events linked to All NEWS2 and SEWS

		NEWS2																				
		0	1	2	3	4	3 single	4 single	5	6	7	8	9	10	11	12	13	14	15	16	17	18	Total SEWS
SEWS	0	200	208	194	150	73	0	1	37	16	5	0	0	0	0	0	0	0	0	0	0	0	884
	1	3	140	162	163	152	39	29	168	108	56	16	4	0	0	0	0	0	0	0	0	0	1040
	2	3	7	55	94	81	30	63	194	188	117	71	37	14	1	2	0	0	0	0	0	0	957
	3	0	2	3	23	55	15	31	135	159	189	130	80	32	16	7	1	0	0	0	0	0	878
	4	0	0	0	2	5	0	12	40	89	96	108	118	62	47	14	1	1	0	0	0	0	595
	5	0	0	0	0	0	0	0	6	24	40	60	81	88	63	23	13	7	2	0	0	0	407
	6	0	0	0	0	0	0	0	0	2	7	24	50	54	54	23	14	5	3	0	0	0	236
	7	0	0	0	0	0	0	0	0	0	1	3	17	24	31	30	17	11	6	1	0	2	143
	8	0	0	0	0	0	0	0	0	0	2	1	5	3	11	14	19	14	11	4	1	0	85
	9	0	0	0	0	0	0	0	0	0	0	0	0	2	1	4	10	5	6	6	0	0	34
	10	0	0	0	0	0	0	0	0	0	0	0	1	0	0	4	2	3	4	3	1	1	19
	11	0	0	0	0	0	0	0	0	0	0	0	0	0	0	0	0	1	0	3	0	0	4
	12	0	0	0	0	0	0	0	0	0	0	0	0	0	0	0	1	1	0	0	0	0	2
	13	0	0	0	0	0	0	0	0	0	0	0	0	0	0	0	0	0	0	0	0	0	0
	Total	206	357	414	432	366	84	136	580	586	513	413	393	279	224	121	78	48	32	17	2	3	5284
	Cum Total	206	563	977	1493	1995			2575	3161	3674	4087	4480	4759	4983	5104	5182	5230	5262	5279	5281	5284	

Of the 220 that NEWS2 = 3 in any single parameter 12 (5.4%) triggered the lowest SEWS threshold ≥4
Of the 580 that NEWS2 = 5, 46 triggered (7.9%) the lowest SEWS threshold ≥4
Of the 586 that NEWS2 = 6, 115 triggered (19.6%) the lowest SEWS threshold ≥4
5284 outcome events are recorded because every NEWS2 is treated independently and linked to an outcome event over the subsequent 24 hours.
Cum Total Is the cumulative total at or below the NEWS2 score *with an event* used to calculate the odds ratio of the composite outcome event when the Index NEWS2 ≥ threshold score *vs.* < threshold score (Fig S3b).

For peer review only

Supplementary Table S4 bed occupancy by the in-patient population at midday

Group	Number of Beds occupied at 12.00[a]
**Patients eligible for NEWS2 analysis**	
emergency admission in hospital < 24 hours at 12.00	98 ± 15
emergency admission in hospital ≥24 hours at 12.00	632 ± 31
elective admission in hospital <24 hours at 12.00	150 (IQR 53-162)
elective admission in hospital ≥24 hours at 12.00	198 ± 21
**Patients not eligible for NEWS2 analysis**	
ICU	72 (IQR: 67-76)
DNACPR	170 ± 15

Supplementary Table 4 legend

Number of beds occupied at midday for the 273 days between 01/11/2018 and 31/7/2019.

[a] Mean ± standard deviation for normally distributed groups. Median and interquartile range if not normally distributed. (Elective admission in hospital <24 hours included day case admissions which vary significantly with the day of the week). In those eligible for NEWS2 analysis, weekday bed occupancy = 1096 (median; IQR: 1079-1113) and weekend bed occupancy = 949 (median; IQR: 937-976). Across all days, the mean daily bed occupancy at midday in those eligible for NEWS2 analysis = 1046.

For peer review only

STROBE Statement—Checklist of items that should be included in reports of ***cohort studies***

	Item No	Recommendation	Response
**Title and abstract**	1	(*a*) Indicate the study's design with a commonly used term in the title or the abstract	Yes. Pg 3
		(*b*) Provide in the abstract an informative and balanced summary of what was done and what was found	Yes. Pg 3
**Introduction**			
Background/rationale	2	Explain the scientific background and rationale for the investigation being reported	Yes. Pg 5
Objectives	3	State specific objectives, including any prespecified hypotheses	Yes. Pg 5
**Methods**			
Study design	4	Present key elements of study design early in the paper	Yes. Pg 6
Setting	5	Describe the setting, locations, and relevant dates, including periods of recruitment, exposure, follow-up, and data collection	Yes. Pg 6
Participants	6	(*a*) Give the eligibility criteria, and the sources and methods of selection of participants. Describe methods of follow-up	Yes. Pg 6-7
		(*b*) For matched studies, give matching criteria and number of exposed and unexposed	N/A
Variables	7	Clearly define all outcomes, exposures, predictors, potential confounders, and effect modifiers. Give diagnostic criteria, if applicable	Yes Pg 6-7
Data sources/ measurement	8*	For each variable of interest, give sources of data and details of methods of assessment (measurement). Describe comparability of assessment methods if there is more than one group	Yes Pg 6-7
Bias	9	Describe any efforts to address potential sources of bias	Yes Pg 6-7
Study size	10	Explain how the study size was arrived at	Yes. Pg 6-7
Quantitative variables	11	Explain how quantitative variables were handled in the analyses. If applicable, describe which groupings were chosen and why	Yes. Pg 6-7
Statistical methods	12	(*a*) Describe all statistical methods, including those used to control for confounding	Yes Pg 8-9
		(*b*) Describe any methods used to examine subgroups and interactions	Yes Pg 8-9
		(*c*) Explain how missing data were addressed	Yes Pg 8-9
		(*d*) If applicable, explain how loss to follow-up was addressed	N/A
		(*e*) Describe any sensitivity analyses	Yes Pg 8-9
**Results**			
Participants	13*	(a) Report numbers of individuals at each stage of study—eg numbers potentially eligible, examined for eligibility, confirmed eligible, included in the study, completing follow-up, and analysed	Yes Pg 10
		(b) Give reasons for non-participation at each stage	Yes Pg 10
		(c) Consider use of a flow diagram	Yes Fig 1
Descriptive data	14*	(a) Give characteristics of study participants (eg demographic, clinical, social) and information on exposures and potential confounders	Yes Table 1
		(b) Indicate number of participants with missing data for each variable of interest	Yes Figure 1
		(c) Summarise follow-up time (eg, average and total amount)	Yes Pg 10
Outcome data	15*	Report numbers of outcome events or summary measures over time	Yes Pg 10
Main results	16	(*a*) Give unadjusted estimates and, if applicable, confounder-adjusted estimates and their precision (eg, 95% confidence interval). Make clear	Yes Pg 11 - 12

For peer review only - http://bmjopen.bmj.com/site/about/guidelines.xhtml

		which confounders were adjusted for and why they were included	
		(*b*) Report category boundaries when continuous variables were categorized	Yes Pg 11 - 12
		(*c*) If relevant, consider translating estimates of relative risk into absolute risk for a meaningful time period	Yes Pg 11 -1 2
Other analyses	17	Report other analyses done—eg analyses of subgroups and interactions, and sensitivity analyses	Yes Pg 11 - 12
**Discussion**			
Key results	18	Summarise key results with reference to study objectives	Yes. Pg 14
Limitations	19	Discuss limitations of the study, taking into account sources of potential bias or imprecision. Discuss both direction and magnitude of any potential bias	Yes Pg 16
Interpretation	20	Give a cautious overall interpretation of results considering objectives, limitations, multiplicity of analyses, results from similar studies, and other relevant evidence	Yes Pg 16
Generalisability	21	Discuss the generalisability (external validity) of the study results	Yes Pg 16
**Other information**			
Funding	22	Give the source of funding and the role of the funders for the present study and, if applicable, for the original study on which the present article is based	Yes Pg 17

*Give information separately for exposed and unexposed groups.

Note: An Explanation and Elaboration article discusses each checklist item and gives methodological background and published examples of transparent reporting. The STROBE checklist is best used in conjunction with this article (freely available on the Web sites of PLoS Medicine at http://www.plosmedicine.org/, Annals of Internal Medicine at http://www.annals.org/, and Epidemiology at http://www.epidem.com/). Information on the STROBE Initiative is available at http://www.strobe-statement.org.

For peer review only
